# Identification of a Unique Cytotoxic Thieno[2,3-c]Pyrazole Derivative with Potent and Selective Anticancer Effects In Vitro

**DOI:** 10.3390/biology11060930

**Published:** 2022-06-18

**Authors:** Jessica D. Hess, Luca H. Macias, Denisse A. Gutierrez, Karla Moran-Santibanez, Lisett Contreras, Stephanie Medina, Paulina J. Villanueva, Robert A. Kirken, Armando Varela-Ramirez, Manuel L. Penichet, Renato J. Aguilera

**Affiliations:** 1 Department of Biological Sciences and Cellular Characterization and Biorepository Core Facility, Border Biomedical Research Center, The University of Texas at El Paso (UTEP), El Paso, TX 79902, USA; jdhess@miners.utep.edu (J.D.H.); lhmacias@miners.utep.edu (L.H.M.); dagutierrez5@utep.edu (D.A.G.); karla.moransn@uanl.edu.mx (K.M.-S.); lcontreras4@miners.utep.edu (L.C.); smedina8@miners.utep.edu (S.M.); pjvillanueva@miners.utep.edu (P.J.V.); rkirken@utep.edu (R.A.K.); avarela2@utep.edu (A.V.-R.); 2 Division of Surgical Oncology, Department of Surgery and Department of Microbiology, Immunology and Molecular Genetics, The Molecular Biology Institute, AIDS Institute, Jonsson Comprehensive Cancer Center, The University of California, Los Angeles, CA 90095, USA; penichet@mednet.ucla.edu

**Keywords:** thienopyrazole, anticancer drug discovery, leukemia, cytotoxicity, differential nuclear staining, apoptosis, kinase activity regulation, microtubule disruption, mitotic spindle organization

## Abstract

**Simple Summary:**

Despite their documented antitumor effects, thienopyrazole-based compounds remain an underexplored class of molecules. In this study, a screening of 2000 novel molecules revealed a unique thienopyrazole derivative, Tpz-1, that elicited potent and selective programmed cell death in human blood, breast, colon, and cervical cancer cell lines when compared to non-cancerous human fibroblast (Hs27) cells. Furthermore, in HL-60 leukemia cells, Tpz-1 interfered with components of signaling pathways and the cytoskeleton that are important to cell shape, internal organization, growth, and division. These findings encourage the continued investigation and development of Tpz-1 and other thienopyrazole derivatives to target and treat cancers.

**Abstract:**

In recent years, the thienopyrazole moiety has emerged as a pharmacologically active scaffold with antitumoral and kinase inhibitory activity. In this study, high-throughput screening of 2000 small molecules obtained from the ChemBridge DIVERset library revealed a unique thieno[2,3-c]pyrazole derivative (Tpz-1) with potent and selective cytotoxic effects on cancer cells. Compound Tpz-1 consistently induced cell death at low micromolar concentrations (0.19 μM to 2.99 μM) against a panel of 17 human cancer cell lines after 24 h, 48 h, or 72 h of exposure. Furthermore, an in vitro investigation of Tpz-1’s mechanism of action revealed that Tpz-1 interfered with cell cycle progression, reduced phosphorylation of p38, CREB, Akt, and STAT3 kinases, induced hyperphosphorylation of Fgr, Hck, and ERK 1/2 kinases, and disrupted microtubules and mitotic spindle formation. These findings support the continued exploration of Tpz-1 and other thieno[2,3-c]pyrazole-based compounds as potential small-molecule anticancer agents.

## 1. Introduction

Small-molecule agents have become a mainstay of targeted cancer treatment in the past 20 years. Unlike macromolecule drugs, small molecules can penetrate cells and bind a wide array of intra- or extracellular targets; additionally, they can be selective or broad-spectrum agents [1,2,3].

**Figure 1:**
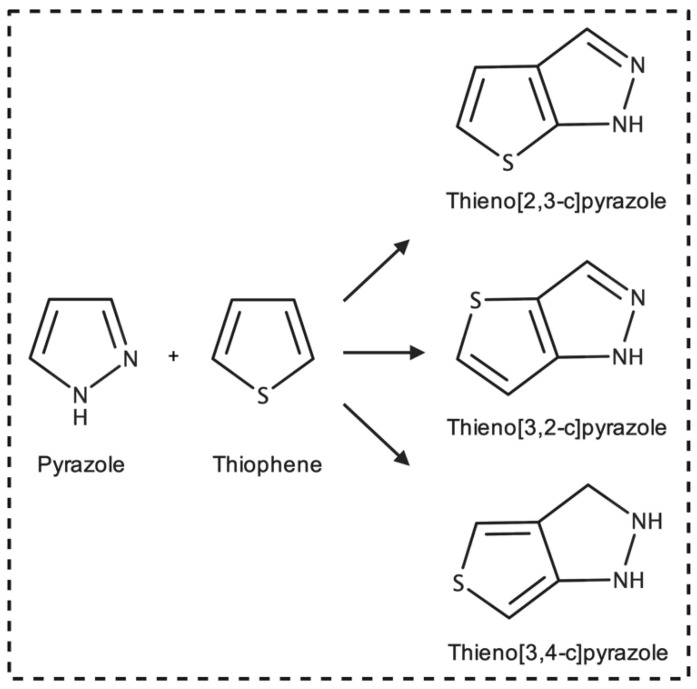
Chemical structures of pyrazole, thiophene, and thienopyrazoles.

Pyrazoles and thiophenes are well-studied pharmacophores known for their diverse and remarkable biological activity, particularly in cancer, and are common scaffolds in small-molecule drug design [4,5]. Pyrazoles are 5-membered heterocycles that contain three carbon atoms and two adjacent nitrogen (Figure 1). Pyrazole derivatives have been approved to treat various leukemias, lymphomas, and other cancers in the United States and China [3]. In addition, numerous experimental pyrazoles have shown antiproliferative activity and apoptosis against several cancer types in vitro [6,7,8]. In contrast, thiophene is a 5-membered heterocycle with four carbon atoms and one sulfur (Figure 1). Thiophene derivatives are also prominent in drug design and are indicated to treat a variety of cancers and other conditions in the U.S. and China [3,9].

Thienopyrazoles are a bicyclic combination of the pyrazole and thiophene moieties and have been endorsed as antiproliferative, antiviral, antimicrobial, and anti-inflammatory agents [10,11,12]. There exist three isomers of thienopyrazole: thieno[2,3-c]pyrazole, thieno[3,2-c]pyrazole, and thieno[3,4-c]pyrazole (Figure 1) [12].

**Figure 2:**
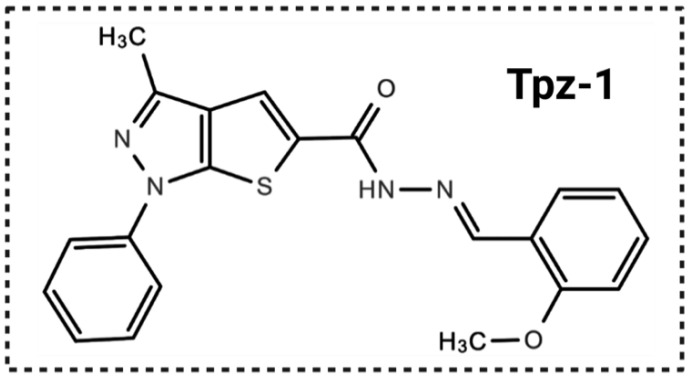
Chemical structure of thieno[2,3-c]pyrazole derivative Tpz-1 (N’-(2-methoxybenzylidene)-3-methyl-1-phenyl-1H-thieno[2,3-c]pyrazole-5-carbohydrazide; MW: 390 g/mol).

Thieno[2,3-c]pyrazoles are less-explored than the other isomers, despite growing evidence detailing their significant phosphodiesterase 7A (PDE7A) [13,14], purinergic receptor P2X3 [15], non-receptor tyrosine kinase ABL [16], Aurora kinase (AURK), insulin-like growth factor type 1 receptor (IGF-1R), and cyclin-dependent kinase 2 (CDK2) inhibition [17,18]. Tpz-1 (Figure 2) is a novel small-molecule thieno[2,3-c]pyrazole derivative that we recently discovered after screening 2000 compounds for novel anticancer agents within the ChemBridge DIVERset library.

Formally named N’-(2-methoxybenzylidene)-3-methyl-1-phenyl-1H-thieno[2,3-c]pyrazole-5-carbohydrazide, we found that this compound was also identified as cytotoxic and a mitotic inhibitor in two previous studies under the aliases 5248881 [19] and MTPC [20]. However, the molecular mechanism by which this unique compound induces cell death was not explored.

Leukemias are a leading cancer type in children and adults [21,22]. In addition, secondary and refractory acute leukemias are known to develop from prior exposure to cytotoxic therapies [23,24,25,26]. Despite recent advances, a subset of patients continue to die from leukemia or complications resulting from treatment [27]. Consequently, we set out to partially elucidate how Tpz-1 potently induces death in acute leukemia cells in vitro.

## 2. Materials and Methods

### 2.1. Cell Lines and Culture Conditions

In this study, the cytotoxicity of Tpz-1 was evaluated against a panel of sixteen cancer cell lines and one non-tumorigenic human cell line. All cell lines were obtained from the American Type Culture Collection (ATCC, Manassas, VA, USA).

Ten of the cell lines were of blood cancer origin: CCRF-CEM (T lymphoblast, ATCC CRL-2265), HL-60 (promyeloblast, ATCC CCL-240), Ramos (B lymphocyte, ATCC CRL-1596), Jurkat (T lymphocyte, ATCC CRL-2899), MOLT-3 (T lymphoblast, ATCC CRL-1552), NALM6 (lymphocyte, ATCC CRL-3273), MM.1S (B lymphoblast, ATCC CRL-2974), MM.1R (B lymphoblast, ATCC CRL-2975), U266 (B lymphocyte, ATCC TIB-196), RPMI 8226 (B lymphocyte, ATCC CRM-CCL-155); and were cultured in RPMI-1640 medium complete with 10% heat-inactivated fetal bovine serum (FBS), 100 U/mL penicillin, and 100 µg/mL streptomycin. An exception was made for HL-60, which required supplementation with an additional 10% FBS. In addition, two colorectal adenocarcinoma cell lines, COLO 205 (ATCC CCL-222) and HT-29 (ATCC HTB-38), were identically maintained in RPMI-1640 complete medium. Two triple-negative breast adenocarcinoma cell lines, MDA-MB-231 (ATCC CRM-HTB-26) and MDA-MB-468 (ATCC HTB-132), and estrogen-receptor-positive (ER+) cell line MCF7 (ATCC HTB-22) were cultured in DMEM medium complete with 10% FBS, 100 U/mL penicillin, and 100 µg/mL streptomycin; cervical adenocarcinoma cell line HeLa (ATCC CRM-CCL-2) and non-tumorigenic foreskin fibroblast Hs27 (ATCC CRL-1634) cells were also maintained in this medium. Lastly, dopaminergic neuroblastoma cell line SH-SY5Y (ATCC CRL-2266) was cultured in DMEM/F12 medium with 10% FBS and antibiotics added as described above. All cells were cultivated in a 5% CO_2_ humidified 37 °C environment.

Before use in experiments, the viability of each cell culture was quantified by flow cytometry following propidium iodide (PI) staining or visualized by trypan blue exclusion [28]. Only cell populations with ≥95% viability and at 60–75% confluence in the exponential growth phase were seeded into multiwell plates to minimize baseline cell death.

### 2.2. Preparation of Compounds

All compounds described herein were commercially purchased. Experimental compounds from the ChemBridge DIVERset library were obtained pre-diluted to 10 mM in dimethyl sulfoxide (DMSO). This ChemBridge stock of N’-(2-methoxybenzylidene)-3-methyl-1-phenyl-1H-thieno[2,3-c]pyrazole-5-carbohydrazide (C21H18N4O2S; MW 390 g/mol), also known as Tpz-1, was used for the experiments detailed below. Lyophilized reference compounds Paclitaxel and Cytochalasin-D were similarly prepared in DMSO; and hydrogen peroxide (H_2_O_2_) was alternatively diluted in 1× phosphate-buffered saline (PBS).

Aliquots of Tpz-1 and other compound stocks were thawed and diluted with DMSO, or 1× PBS for H_2_O_2,_ to a 100× or 250× working concentration for use in experiments to achieve a final vehicle concentration of 1% *v*/*v* or less.

### 2.3. Initial Library Screening and Compound Selection

Two thousand experimental compounds of the ChemBridge DIVERset library were initially screened against the CCRF-CEM cell line in vitro at a single concentration of 10 μM by differential nuclear staining assay. Any compound which induced >50% cell death at 10 μM was isolated and additionally screened at concentrations ranging from 0.01 to 10 μM to calculate the dose of compound needed to induce 50% cell death.

### 2.4. Differential Nuclear Staining Assay

Differential nuclear staining (DNS) is a live cell imaging assay that uses two nuclear fluorescent dyes to distinguish between live and dead populations based on cell permeability [29]. To achieve this, Hoechst 33342 (blue) is used to stain the nuclei of all cells in a sample, whereas PI (red) is used to distinguish only those with damaged membranes. Thus, cells that co-localize both red and blue signals, exhibiting a magenta color, are defined as the dead/dying population.

For DNS analysis, cells were first seeded in 96-well plates at a density of 10,000 cells/well in 100 μL of complete medium and incubated overnight to facilitate cell attachment and acclimation. The next day, cells were exposed to Tpz-1 concentrations ranging from 0.01 to 10 μM, 1% *v*/*v* DMSO (vehicle control), 1 mM H_2_O_2_ (positive control), or left untreated (UNT) for 24–72 h. Four independent measurements were used for the samples, except for the vehicle control in which eight DMSO-treated independent measurements were used to ensure the average total cell count was representative for each experiment series. One to two hours before the end of treatment, a 1 μg/mL final concentration of Hoechst 33342 and PI was quickly added to each well and incubated until the remaining time elapsed. Four contiguous images, taken in 2 × 2 montages, were captured via GE InCell Analyzer 2000 with a 10x objective from the two fluorescent channels corresponding to Hoechst and PI emission. After analysis, the captured high-content images were segmented using the GE InCell Analyzer Workstation 3.2 software to obtain live and dead cell counts and percentages within each well. Due to technical problems, for the MCF7 cell line, the BD Pathway 855 bioimaging system was used to capture a similar 2 × 2 montages per well, and images were segmented with its associated AttoVision v1.6.2 software (BD Biosciences, Rockville, MD, USA). To account for the lower total cell count in Tpz-1 or H_2_O_2_-treated (Treatment) samples caused by cell destruction (disappearance from count) over the course of incubation, cell death percentages were normalized to the vehicle control (DMSO) average total cell count by the following equation:

$$\frac{\left(\mathrm{DMSO}\;\mathrm{average}\;\mathrm{total}\;\mathrm{cell}\;\mathrm{count}-\mathrm{Treatment}\;\mathrm{total}\;\mathrm{cell}\;\mathrm{count}\right)+\mathrm{Treatment}\;\mathrm{dead}\;\mathrm{cell}\;\mathrm{count}}{\mathrm{DMSO}\;\mathrm{average}\;\mathrm{total}\;\mathrm{cell}\;\mathrm{count}}\times \;100$$

Normalized percentages of dead cells per treatment replicate were subsequently averaged and used to estimate CC_50_ values.

### 2.5. Cytotoxic Concentration 50% and Selective Cytotoxicity Index Values

The cytotoxic concentration 50% (CC_50_) for each cell line indicates the average concentration (μM ± S.D.) needed to kill 50% of the cell population at a given time point. From the percentage of cell death for each technical replicate at a concentration above and below 50% cell death, CC_50_ for each cell line was calculated via linear interpolation and averaged to obtain the reported values [7,30,31].

To measure Tpz-1′s cancer selectivity in vitro, selective cytotoxicity index (SCI) values were calculated by dividing the CC_50_ of non-tumorigenic control cell line Hs27 by the CC_50_ of individual cancer cell lines at the same incubation time [32].

### 2.6. AnnexinV-FITC/PI Assay

To determine if Tpz-1 stimulates apoptotic or necrotic cell death, HL-60 acute myeloid leukemia or CCRF-CEM T lymphoblast leukemia cells were transferred to a 24-well plate at a seeding density of 100,000 cells in 1 mL complete culture media. After overnight incubation, cells were treated with the cell line’s 24 h CC_50_ or 2xCC_50_ of Tpz-1 for 24 h. Also, 0.1% *v*/*v* DMSO, 100 μM H_2_O_2_, and untreated cells were used as the vehicle, positive, and negative controls. All results, experimental points, and corresponding controls represent three independent determinations. Immediately following treatment, cells were pelleted at a centrifuge pre-cooled to 4 °C for 5 min at 500× *g*. The resulting supernatant was discarded, and cell pellets were resuspended in 103.5 μL of a cold AnnexinV-FITC/PI/1× binding buffer mixture, then chilled on ice while covered for 30 min. Afterward, 300 μL of ice-cold 1× binding buffer was added to each well immediately prior to flow cytometry. Total apoptosis was calculated by summing early apoptotic (AnnexinV-FITC positive/PI negative signal) and late apoptotic (AnnexinV-FITC positive/PI positive) cell populations. The statistical significance of the apoptosis induced by Tpz-1 was evaluated by comparing experimentally treated samples to those of DMSO by *t*-test (*p* = 0.05). Approximately 20,000 events (cells) were acquired per sample. Data analysis was achieved via the flow cytometer’s Kaluza software (Beckman Coulter, Indianapolis, IN, USA).

### 2.7. Caspase Activation Assays

Caspase-3/7 are proteases activated in the execution phase of apoptosis’s intrinsic and extrinsic pathways [33]. Therefore, to further validate that apoptosis is the cell death mechanism of Tpz-1, caspase-3/7 activation (NucView 488 Caspase-3/7 Substrate Assay Kit, Biotium) was measured in compound-treated HL-60 acute myeloid leukemia and CCRF-CEM T cell leukemia cells [34]. The substrate provided in the kit consists of a fluorogenic DNA dye with DEVD moiety that is specific for caspase-3/7. When cleaved by caspase-3/7, the substrate forms a high-affinity nuclear dye that emits a bright green signal detectable by flow cytometry. For this assay, cells were seeded in 24-well plates at a density of 100,000 cells per well in 1 mL complete culture medium and incubated overnight. Cells were subsequently treated with the appropriate 24 h CC_50_ or 2xCC_50_ of Tpz-1 for 6 h (data shown) or 8 h (in Supplementary Materials). Controls were included as previously described. Three independent measurements were analyzed for each experimental and control treatment. At the end of treatment, cells were collected and centrifuged at 262× *g* for 5 min. The supernatant was discarded, and pelleted cells were resuspended in 102.5 µL of 1× PBS and NucView 488 Caspase-3/7 substrate mixture (5 µM final concentration), then covered and incubated at room temperature for 45 min. After, 300 µL of 1× PBS was added, and a flow cytometer was used to analyze samples immediately. Cells emitting green fluorescence were defined as apoptotic cells with active caspase-3/7. Approximately 20,000 events (cells) were acquired per sample, and the Beckman Coulter Kaluza software was used for data analysis.

Caspase-8, in contrast, is an initiator of the extrinsic pathway of apoptosis and its activation directly promotes the cleavage of downstream executioner caspase-3/7 [33,35]. Hence, to evaluate if Tpz-1 stimulates extrinsic apoptosis, we assessed the activation of caspase-8 via substrate assay kit following the manufacturer’s protocol (Abcam; Ab65614) to stain HL-60 cells after 4 h treatment with the 24 h CC_50_ or 2xCC_50_ of our compound. In this experiment, the caspase-8-specific inhibitor IETD-FMK is conjugated to FITC and emits fluorescence that is detectable by flow cytometry when bound to active caspase-8. In addition, cell seeding, controls, data acquisition, and data analysis were performed in a manner consistent with our other cytometric experiments.

### 2.8. Reactive Oxygen Species Assay

Reactive oxygen species (ROS) can be an indicator of mitochondrial dysfunction in intrinsic apoptosis [36]. Thus, we measured the accumulation of ROS was by 6-carboxy-2′,7′-dichlorodihydrofluorescein diacetate (carboxy-H2DCFDA) reagent (Invitrogen, C400) [37]. In this experiment, the acetate groups of nonfluorescent carboxy-H2DCFDA are cleaved by intracellular esterases and oxidation to form the green fluorescing product 2′,7′-dichlorofluorescein (DCF). HL-60 acute myeloid leukemia or CCRF-CEM T cell leukemia cells were each distributed into 24-well plates at a seeding density of 100,000 cells/well in 1 mL complete medium and treated with the appropriate 24 h CC_50_ or 2xCC_50_ of Tpz-1 or controls, as previously mentioned, for 18 h [37]. Three independent measurements were evaluated for all experimental and control treatments. Post-treatment, samples were collected and centrifuged for 5 min at 262× *g*, then resuspended in a pre-warmed mixture of 1× PBS and carboxy-H2DCFDA at a final concentration of 10 µM dye. Cells were incubated for 1 h at 37 °C in a 5% CO_2_ humidified environment, centrifuged at 262× *g* for 5 min, then resuspended in 500 µL PBS, and analyzed by flow cytometer after a 30-min recovery period. Approximately 20,000 cell events were obtained and analyzed per sample, and data analysis was achieved as mentioned in the cytometric analyses described above.

### 2.9. Mitochondrial Depolarization Assay

To monitor the mitochondrial integrity of cells exposed to Tpz-1, changes in mitochondrial membrane potential were studied via MitoProbe JC-1 Assay Kit (Molecular Probes, M34152). 5′,6,6′-tetrachloro-1,1′,3,3′-tetraethylbenzimidazolylcarbocyanine iodide (JC-1) is a fluorescent cationic probe capable of accumulating inside mitochondria. In healthy mitochondria, the JC-1 dye aggregates to spontaneously form red fluorescent complexes, however, in damaged mitochondria, the JC-1 dye cannot accumulate enough for these complexes to develop, and the dye alternatively emits green fluorescence [38,39]. The red/green fluorescence ratio of JC-1 is thus a reliable indicator of mitochondrial integrity. For this assay, HL-60 or CCRF-CEM cells were seeded in 24-well plates at a density of 100,000 cells/well in 1 mL complete culture media and were treated with the 24 h CC_50_ or 2xCC_50_ of Tpz-1 and controls (as previously described) for 5 h. Three independent measurements were analyzed per cell treatment. When the incubation time expired, cells were collected and centrifuged at 262× *g* for 5 min, then resuspended in 500 μL 1× PBS containing 2 μM JC-1 dye. Cells were stored at 37 °C in a 5% CO_2_ humidified incubator for 30 min, then washed with warm PBS before analysis by flow cytometer. Green fluorescing cells were an indication of depolarized mitochondria within a given sample. Approximately 20,000 events were acquired per sample, and data analysis was achieved as mentioned for other cytometric analyses.

### 2.10. Cell Cycle Analysis

To determine the effect of Tpz-1 on the cell cycle, the DNA content of individual cells was quantified by nuclear isolation medium (NIM)-DAPI staining (NPE Systems, Inc./Beckman Coulter). The NIM-DAPI reagent simultaneously permeabilizes plasma membranes and stains cellular DNA with a violet-excited DNA-intercalating agent (DAPI). A cell’s DAPI signal is directly proportional to the amount of cellular DNA such that G0/G1 < S < G2/M, hence DAPI fluorescence intensity was used to quantify the percentage of cells in each phase of the cycle for each sample. For this experiment, asynchronously cultured HL-60 and CCRF-CEM cells were separately seeded in 24-well plates at a density of 50,000 cells/well in 1 mL of complete culture media, incubated overnight, and treated with the appropriate 24 h ¼ CC_50_ or ½ CC_50_ of Tpz-1 for 72 h. These concentrations were selected for consistency with our other cytometric analyses. 0.1% *v*/*v* DMSO, 100 μM H_2_O_2_, and untreated controls were also included. All treatments were assessed using three independent measurements. After treatment, cells were collected and centrifuged at 262× *g* for 5 min. The supernatant was discarded, cell pellets were resuspended in a mixture of 100 μL 1× PBS and 200 μL NIM-DAPI solution, then quickly analyzed via flow cytometer. Fluorescent signal was captured using an FL9 detector and a 405 nm laser. Approximately 20,000 events (cells) were analyzed per sample, with data analysis achieved as previously mentioned.

### 2.11. Phospho-Kinase Arrays

The phosphorylation of tyrosine-protein Src family and mitogen-activated protein (MAP) kinases were assessed on Tpz-1 treated vs. untreated cells via Multiplex Luminex assay kits. The phosphorylation status of MAP kinases CREB, JNK, NFĸB, p38, ERK 1/2, Akt, p70S6k, STAT3, and STAT5 were detected via the Milliplex MAP human multi-pathway signaling network kit (Millipore). Similarly, phosphorylation of Src kinases Src, Fyn, Yes, Lck, Lyn, Fgr, Blk, and Hck were detected using the Milliplex MAP 8-Plex human Src family kinase kit (Millipore). In these experiments, 5 × 10^6^ HL-60 cell samples were collected in microcentrifuge tubes and treated with a sub-CC_50_ dose, 0.01 μM or 0.1 μM, of Tpz-1 and incubated for 3 h at 37 °C. The cells in each sample were lysed, their protein concentrations determined, and Multiplex Luminex assays were performed following the same procedure as the experimental compound P3C [7]. Acquisition and data analysis were performed using Luminex xPONENT 3.1 software.

### 2.12. Confocal Microscopy

To assess the effect of Tpz-1 on cytoskeletal architecture, non-tumorigenic Hs27 and cervical adenocarcinoma HeLa cell lines were utilized [31]. Cells were detached from their vessels and separately seeded into thin-bottomed 96-well plates at a density of 2.5 × 10^3^ cells in 100 μL complete medium and incubated at 37 °C overnight to promote reattachment. The next day, a 10 µM dose of Tpz-1 was applied to Hs27 cells and a 0.19 µM dose to HeLa cells for 4 h and 24 h, respectively. Additionally, vehicle (1% *v*/*v* DMSO), actin polymerization inhibitor (5 μg/mL Cytochalasin-D), tubulin stabilizer (1 μM Paclitaxel), and untreated controls were included.

After incubation, 100 μL of freshly prepared 8% formaldehyde was added directly to each well as a fixative solution, and the plate was incubated at room temperature for 20 min. Next, the liquid in each well was removed, and 200 μL of 0.1% *v*/*v* Tween 20 in 1× PBS was added for washing/permeabilization and then incubated for 10 min at room temperature. After washing cells twice more, the liquid was removed from each well and 200 μL of 5% *w*/*v* bovine serum albumin (BSA) dissolved in Tris-buffered saline with 0.5% *v*/*v* Tween 20 (TBS-T) was added for blocking purposes. Samples were then incubated at room temperature and on a rocking platform for 1 h with the blocking solution to minimize non-specific binding. Afterward, samples were triple-stained with 50 μL of 0.1% *v*/*v* Tween 20 in 1× PBS solution containing 5 μg/mL DAPI to visualize nuclei, 0.165 μM Alexa Fluor 568-conjugated phalloidin for actin microfilaments (F-actin), and 0.5 μg/mL Alexa Fluor 488-conjugated anti-α-tubulin monoclonal antibody for microtubules (polymerized tubulin). The stained cells were protected from light and placed on a rocking platform for 1 h at room temperature. Next, samples were washed three times with 200 μL of 0.1% *v*/*v* Tween 20 permeabilization solution. After the final wash, cells were kept in a fresh 200 μL of 0.1% *v*/*v* Tween 20. Images of stained cells were subsequently captured on three fluorescent channels (DAPI, Alexa-568, and Alexa-488) with a Zeiss LSM-700 confocal microscope equipped with an EC Plan-Neofluar 40×/1.30 oil DIC objective. A 1-Airy Unit (AU) pinhole setting was consistently utilized for each channel. For image acquisition and analysis, the ZEN 2009 software was used (Zeiss, New York, NY, USA).

### 2.13. Statistical Analysis

All flow cytometry experimental points are representative of three independent measurements, whereas, for differential nuclear staining, four independent measurements were performed. Eight independent measurements were used to obtain the total cell count for vehicle-treated samples in each DNS assay. Experimental values are reported as the average of independent measurements per treatment with corresponding standard deviation. Statistical significance was determined by two-tailed paired Student’s *t*-tests (*p* = 0.05) comparing treatment samples to vehicle controls; *p*-values ≤ 0.05 were considered significant. Significance values are reported as * *p* < 0.05, ** *p* < 0.01, and *** *p* < 0.001.

## 3. Results

### 3.1. Drug Screening Identified a Thienopyrazole Derivative (Tpz-1) with Potent Cytotoxicity against Several Cancer Cell Lines

We initiated a high-throughput screening project of 2000 compounds from the ChemBridge DIVERset chemical library in search of novel cancer-killing compounds. Cytotoxicity of each compound was first explored via DNS assay against the acute lymphoblastic leukemia cell line CCRF-CEM, and compounds that elicited 50% or greater cell death after 48 h of exposure were isolated for further analysis. Tpz-1 (Figure 2) emerged as the most attractive candidate, with a CC_50_ of 0.25 μM toward the CCRF-CEM cell line, and. was thus retested for its ability to induce cell death in other cell types.

### 3.2. Tpz-1 Displays Highly Selective Cytotoxicity against Leukemia and Lymphoma Cell Lines

To elucidate the potential selectivity of Tpz-1 toward different cell types, cytotoxicity was examined against a panel of human cell lines derived from a variety of cancerous blood and tissues.

The cytotoxicity of Tpz-1 toward individual cell lines was assessed between 24–72 h of exposure through the DNS assay. Cells that grow in suspension were evaluated generally after 48 h, whereas adherent cells were incubated for 72 h due to their slower doubling time. In 14 cancer cell lines, the CC_50_ of Tpz-1 was below 1 µM (Table 1). Additionally, Tpz-1 was noticeably less potent toward non-cancerous Hs27 cells.

From the CC_50_ values calculated at 48 h and 72 h, selective cytotoxicity index (SCI) scores were calculated by dividing the CC_50_ of Hs27 cells by the CC_50_ of individual cancer cell lines (Table 2). Very high selectivity (SCI ≥ 20.37) was observed in CCRF-CEM, Ramos, NALM6, HL-60, and SHSY-5Y cell lines, and scores were also favorable (SCI ≥ 2.0) across the other cancer cell lines we tested. Interestingly, the SCI for hematopoietic cells averaged 13.82; thus, in vitro specificity of Tpz-1 toward blood cancers exists. Of the hematologic cancer cell lines in our panel, Tpz-1 was most sensitive and selective toward leukemias (SCI = 15.48). As a result of this selectivity, the cytotoxicity of Tpz-1 against HL-60 and CCRF-CEM cells was also evaluated via DNS at 24 h, and these cell lines were chosen as our in vitro models to assess Tpz-1′s ability to induce apoptosis.

### 3.3. Tpz-1 Triggers Movement of Phosphatidylserine to the Outer Plasma Membrane

**Figure 3:**
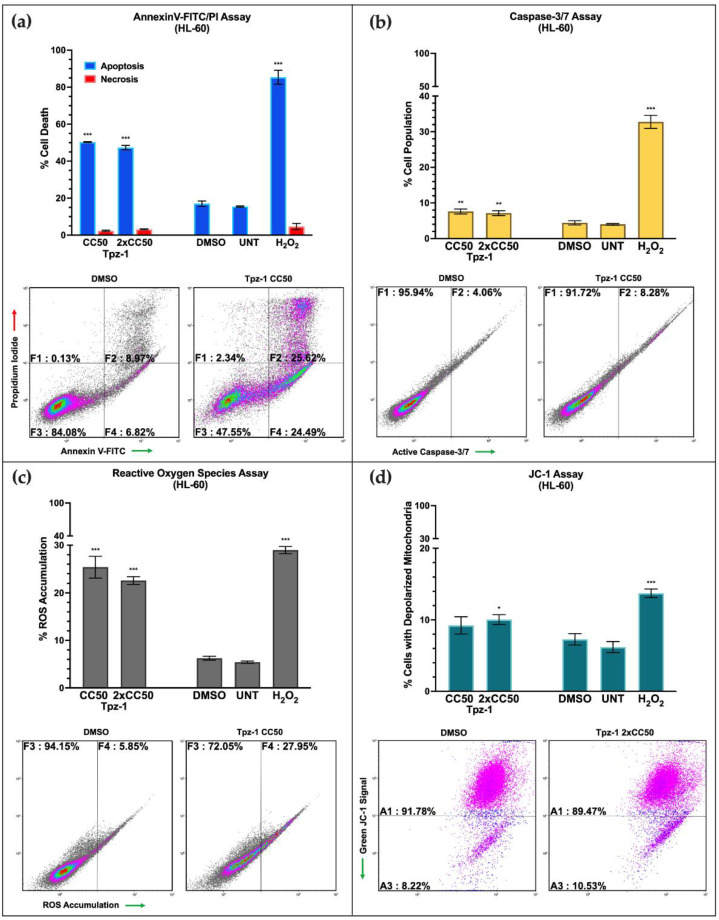
The intrinsic apoptosis pathway mediates Tpz-1-induced cell death. (**a**) Analysis of phosphatidylserine externalization by flow cytometry showed apoptosis induction in HL-60 cells after 24 h of exposure to CC_50_ (0.95 μM; *p* < 0.00001) and 2xCC_50_ (1.9 μM; *p* < 0.00001) concentrations of Tpz-1. The percentages displayed represent total (early and late) apoptosis values. (**b**) Flow cytometry analysis indicated that Tpz-1 CC_50_ (0.95 μM; *p* = 0.00646) and 2xCC_50_ (1.9 μM; *p* = 0.005941) concentrations induced significant caspase-3/7 activation after 6 h of incubation in HL-60 cells. Positive fluorescence for NucView 488 Caspase-3/7 substrate indicated caspase-3/7 activation. (**c**) Tpz-1 causes reactive oxygen species accumulation in HL-60 cells at CC_50_ (0.95 μM; *p* = 0.000139) and 2xCC_50_ (1.9 μM; *p* < 0.00001) concentrations. ROS was quantified using the carboxy-H_2_DCFDA reagent. (**d**) Tpz-1 disrupted mitochondrial membrane potential in HL-60 cells after 5 h of incubation. A modest but significant increase in depolarized mitochondria was seen in cells treated with Tpz-1 2xCC_50_ (1.9 μM; *p* = 0.029193). Mitochondrial depolarization was quantified using the JC-1 dye; an increase in green fluorescent signal denoted loss of membrane potential. (**a**–**d**) Cells were treated with the 24 h CC_50_ (0.95 μM) or 2xCC_50_ (1.9 μM) for the HL-60 cell line, and 1% *v*/*v* DMSO or 1 mM H_2_O_2_ as vehicle and positive controls for cytotoxicity, respectively. Untreated (UNT) controls were also included. The percentages and standard deviations represent the average of three technical replicates. Representative density plots for each experiment are shown. Statistical analysis was achieved by two-tailed Student’s paired *t*-test, and the asterisk annotations represent the statistical significance of the treatments against the vehicle control; (*) *p* < 0.05, (**) *p* < 0.01, (***) *p* < 0.001.

Tpz-1 treatment caused significant translocation of phosphatidylserine (PS) to the outer leaflet of cell plasma membranes after 24 h, a hallmark event of apoptosis [40]. To distinguish between apoptotic and necrotic cell death, we performed an Annexin V-FITC/PI assay. The Annexin V protein (~36.8 kDa) is a high-affinity ligand of PS, which, when conjugated to the fluorophore fluorescein-5-isothiocyanate (FITC), can be readily detected by flow cytometry when bound to PS. In this experiment, cells in the early stages of apoptosis were distinguished by their emission of FITC signal. In contrast, membrane-compromised late-stage apoptotic and necrotic cells were detected by propidium iodide (PI); those positive for both FITC and PI were considered apoptotic, and those only positive for PI were deemed necrotic. Our results showed significant PS translocation after 24 h of exposure to Tpz-1 at the predicted 24 h CC_50_ and 2xCC_50_ concentrations (HL-60 *p* < 0.001, Figure 3a; CCRF-CEM CC_50_ *p* < 0.05 and 2xCC_50_ *p* < 0.001, Figure S1a), suggesting that the primary mode of cell death for this compound is apoptosis.

### 3.4. Tpz-1 Induces Caspase-Dependent Apoptosis

To support our evidence that Tpz-1 induces apoptosis, caspase-3/7 activation was measured in CCRF-CEM and HL-60 cells by flow cytometry. Caspase-3/7 is an integral part of the intrinsic and extrinsic pathway machinery; therefore, cells were treated with the CC_50_ or 2xCC_50_ of Tpz-1 for 6 h (both cell lines) or 8 h (CCRF-CEM only) to sufficiently stimulate apoptosis before staining for cleaved caspase-3/7 and to optimize fluorophore signal intensity. Our results revealed a modest but significant activation of caspase-3/7 in the two Tpz-1 treatments (HL-60 *p* < 0.01; CCRF-CEM *p* < 0.05) compared to vehicle-treated control samples at 6 h (Figure 3b and Figure S1b). Decreased caspase-3/7 activation was observed at the 8 h time point in CCRF-CEM cells (Figure S2), indicating that peak caspase-3/7 activation occurs closer to 6 h after treatment. Similarly, to determine if Tpz-1 stimulates extrinsic apoptosis, we evaluated for the activation of the key initiator, caspase-8; however, we did not detect an increase in activity after 4 h of treatment (Figure S3). Together, this data puts forth intrinsic apoptosis as a probable mode of cell death inflicted by Tpz-1.

### 3.5. Tpz-1 Causes Cell Stress through an Accumulation of Reactive Oxygen Species

Reactive oxygen species (ROS) indicate cellular homeostatic disturbance, and their accumulation promotes apoptosis [41]. To assess for physiological damage inflicted by Tpz-1, ROS accumulation was quantified with the carboxy-H_2_DCFDA reagent and analyzed by flow cytometry. Accumulation of ROS in HL-60 and CCRF-CEM cells was assessed after exposure to the 24 h CC_50_ or 2xCC_50_ of Tpz-1 for 18 h. Significant ROS accumulation in Tpz-1 treated samples was evident (*p* < 0.001; Figure 3c and Figure S1c), suggesting the contribution of oxygen radicals to apoptosis.

### 3.6. Tpz-1 Treatment Disrupts Mitochondrial Function

The intrinsic apoptosis pathway is associated with mitochondrial outer membrane permeabilization that drives the release of ROS and pro-apoptotic proteins from mitochondria [36,42]. Therefore, we investigated the loss of mitochondrial membrane integrity using the JC-1 reagent. The JC-1 dye enters and accumulates in functional mitochondria to form red fluorescent complexes. In contrast, in mitochondria that have lost membrane potential, the dye can no longer accumulate and emits green fluorescence. An increase in green emission signal can therefore distinguish compound-induced mitochondrial depolarization. Our analysis revealed a slight but significant depolarization of mitochondria in HL-60 and CCRF-CEM cells after treatment with Tpz-1 (*p* < 0.05) for 5 h (Figure 3d and Figure S1d). This data additionally supports the involvement of the intrinsic pathway of apoptosis.

### 3.7. Tpz-1 Cell Death Is Restricted to Cells in the G2/M Phase

**Figure 4:**
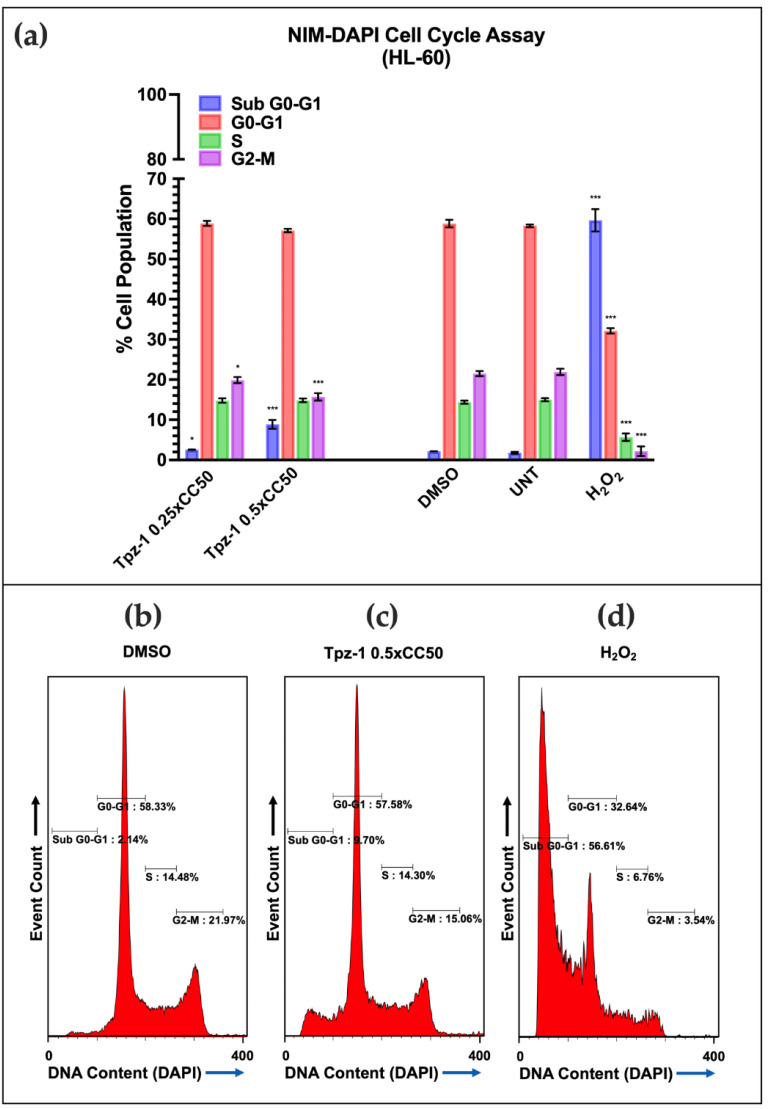
(**a**) Tpz-1 cytotoxicity is G2-M phase-dependent in HL-60 cells after 72 h of exposure to the 24 h 0.25xCC_50_ (0.24 μM; Sub G0-G1 *p* = 0.047421; G2-M *p* = 0.046939) or 0.5xCC_50_ (0.48 μM; Sub G0-G1 *p* = 0.000484; G2-M *p* = 0.000873). NIM-DAPI was used to stain and quantify the DNA content of each sample. Tpz-1-treated cells displayed DNA fragmentation, denoted by an increase of cells in sub G0-G1, that was concurrent with a loss of cells in G2-M. 0.1% *v*/*v* DMSO and 100 µM H_2_O_2_ were utilized as vehicle and positive controls for cytotoxicity, respectively. Untreated (UNT) controls were also included. The percentages and standard deviations represent the average of three technical replicates. (**b**–**d**) Representative histograms for DMSO, 0.5xCC_50_ Tpz-1, and H_2_O_2_ treated samples. Statistical analysis was achieved by two-tailed Student’s paired *t*-test, and the asterisk annotations in each graph represent the statistical significance of the treatments against the vehicle control; (*) *p* < 0.05, (***) *p* < 0.001.

To assess the dependence of Tpz-1 activity on the cell cycle, phase distribution was evaluated in HL-60 and CCRF-CEM cells after treatment with the 24 h ¼xCC_50_ or ½xCC_50_ Tpz-1 for 72 h. Low concentrations of Tpz-1 were used to minimize the interference of fragmented DNA in our assessment. Samples were stained with a nuclear isolation medium containing the fluorophore DAPI (NIM-DAPI) post-incubation. In this assay, the DAPI signal is proportional to the amount of cellular DNA at each phase. Thus, its emission was used to quantify the DNA content in each sample by flow cytometry. As predicted, a significant increase in DNA fragmentation (represented by the sub-G0/G1 phase) was evident in both HL-60 and CCRF-CEM and denoted the apoptotic population in each sample (¼xCC_50_ *p* < 0.05; ½xCC_50_ *p* < 0.001). Although DNA fragmentation was successfully attenuated in HL-60 cells (~10% of the population), the sub-G0/G1 population was twice as large in CCRF-CEM (~20%). A decrease in the G2/M phase was seen in HL-60 cells treated with Tpz-1, revealing an inverse relationship between Tpz-1 treated cells in the sub-G0/G1 and G2/M stages of cell division (Figure 4). These results establish a potential connection between Tpz-1 activity and cells as they prepare for mitosis. However, the overabundant DNA fragmentation in the CCRF-CEM cell line made it difficult to distinguish the different cell populations (Figure S4), and so it remains unclear if this phenomenon is shared or unique to HL-60 cells.

### 3.8. Tpz-1 Interferes with Src and MAP Kinase Function

**Figure 5:**
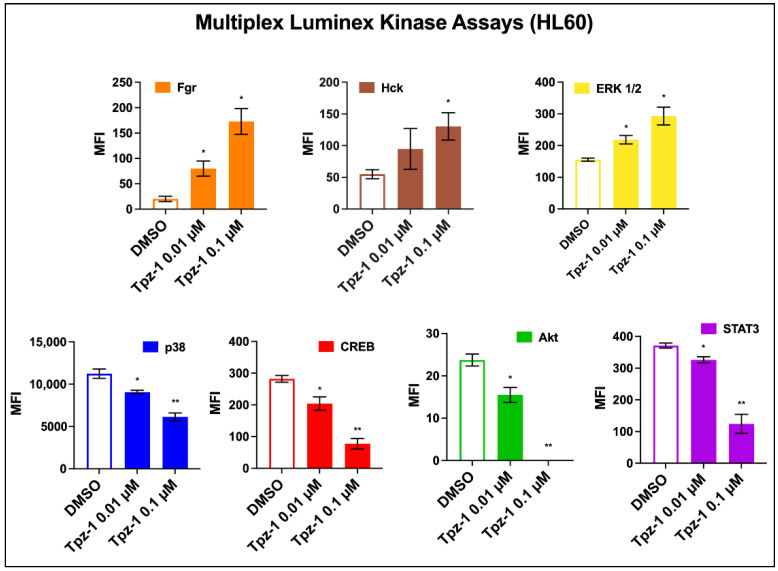
Tpz-1 interferes with Src and MAP pathway signaling in HL-60 cells after 3 h of exposure. Cellular extracts from cells exposed to 0.01 μM Tpz-1, 0.1 μM Tpz-1, or 0.2% *v*/*v* DMSO were analyzed for changes in kinase phosphorylation via antibody/bead-based Luminex xMAP technology. The *y*-axis indicates median fluorescence intensity (MFI) and error bars represent the standard deviations of the average of two technical replicates. Decreased MFI, implying kinase hypophosphorylation was evident in p38 (0.01 µM *p* = 0.034225; 0.1 µM *p* = 0.009669), CREB (0.01 µM *p* = 0.043251; 0.1 µM *p* = 0.004603), Akt (0.01 µM *p* = 0.035648; 0.1 µM *p* = 0.002076), and STAT3 (0.01 µM *p* = 0.037819; 0.1 µM *p* = 0.00785); whereas increased MFI, implying hyperphosphorylation was observed in Fgr (0.01 µM *p* = 0.033102; 0.1 µM *p* = 0.014181), Hck (0.1 µM *p* = 0.0426), and ERK 1/2 (0.01 µM *p* = 0.024866, 0.1 µM *p* = 0.020551). Statistical analysis was achieved by two-tailed Student’s paired *t*-test, and the asterisk annotations in each graph represent statistical significance of the treatments against the vehicle control; (*) *p* < 0.05, (**) *p* < 0.01. Data acquisition and analysis was achieved via xPONENT 3.1 software (Luminex, Austin, TX, USA).

Protein kinases regulate many cellular functions, including proliferation, mitosis, apoptosis, and metabolism. Dysregulation of these processes is frequently oncogenic. Thus, there have been considerable efforts to develop small-molecule kinase inhibitors to treat a variety of cancers. Kinase inhibitors remain a popular target in anticancer drug discovery today, and several have been approved for clinical use in recent years. Studies with other thienopyrazole-derivatives have also shown substantial kinase inhibition [13,16,17,18], prompting the need to assess Tpz-1’s effect on a multi-pathway panel of human kinases. Changes in phosphorylation of Src and MAP kinases were assessed by Multiplex Luminex assay using 0.01 µM or 0.1 µM Tpz-1-treated HL-60 cell lysates. Decreased median fluorescence intensity (MFI) was observed in MAP family kinases p38 (0.01 µM *p* < 0.05, 0.1 µM *p* < 0.01; Figure 5), CREB (0.01 µM *p* < 0.05, 0.1 µM *p* < 0.01; Figure 5), Akt (0.01 µM *p* < 0.05, 0.1 µM *p* < 0.01; Figure 5), and STAT3 (0.01 µM *p* < 0.05, 0.1 µM *p* < 0.01; Figure 5) and implies kinase hypophosphorylation; whereas increased MFI/hyperphosphorylation was evident in Src family kinases Fgr (0.01 µM and 0.1 µM *p* < 0.05; Figure 5), Hck (0.1 µM *p* < 0.05; Figure 5), and MAP kinase ERK 1/2 (0.01 µM and 0.1 µM *p* < 0.05; Figure 5).

### 3.9. Tpz-1 Treatment Impedes Microtubule Organization

To ascertain the effect of Tpz-1 on the cytoskeleton and cell division, non-cancerous Hs27 and cancerous HeLa cell lines were treated and stained to visualize DNA content (DAPI; blue), actin microfilaments (Alexa 568; red), and microtubules (Alexa 488; green). Hs27 cells are large fibroblasts with an elongated shape, making them ideal for observing alterations in cytoskeletal organization; similarly, HeLa’s rapid proliferation and large surface area renders them optimal for observing mitotic spindle changes.

**Figure 6:**
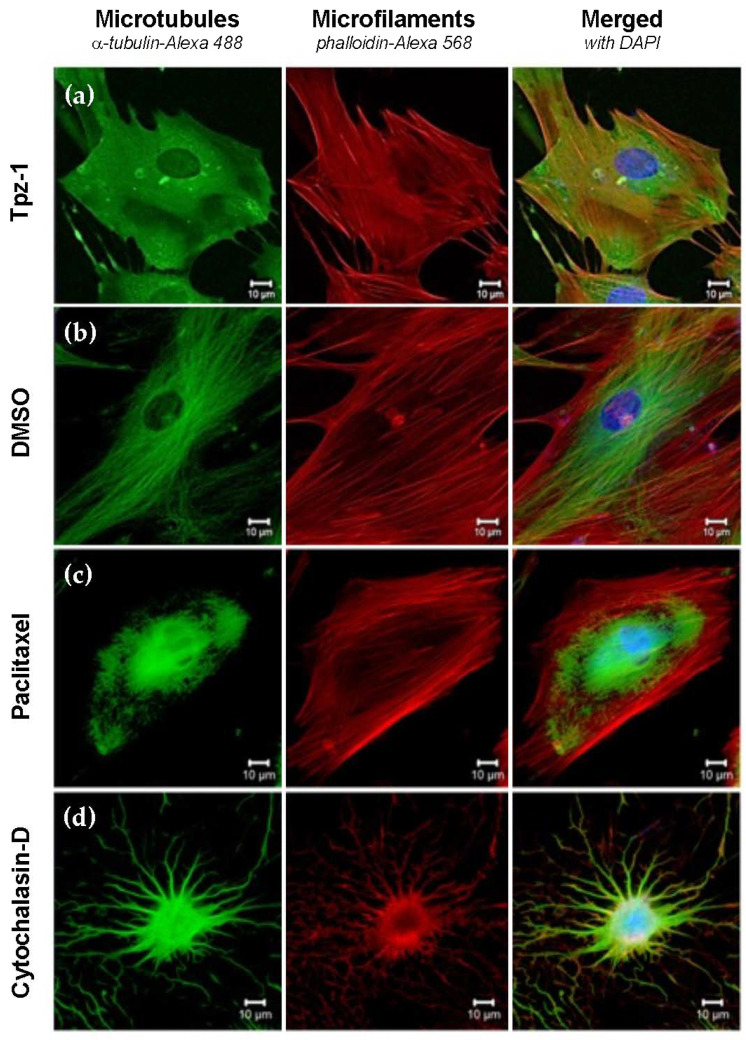
Tpz-1 (10 µM) inhibits tubulin polymerization in Hs27 cells after 4 h of exposure. (**a**) Tpz-1, (**b**) 1% *v*/*v* DMSO, (**c**) 1 µM Paclitaxel, and (**d**) 5 µg/mL Cytochalasin-D treated cells were stained with α-tubulin-Alexa-488 (microtubules), phalloidin-Alexa-568 (microfilaments), and DAPI (nucleus) prior to immunofluorescent analysis by confocal microscopy. DMSO was included as vehicle control, and Paclitaxel and Cytochalasin-D as microtubule and microfilament disruptive agents, respectively.

**Figure 7:**
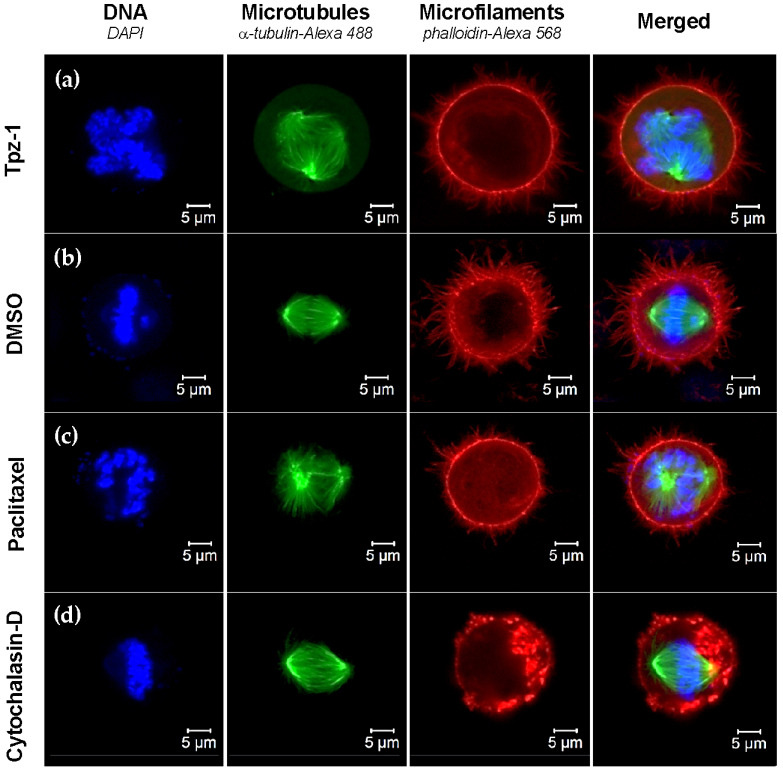
Tpz-1 (0.19 µM) disrupts spindle formation and polarity in HeLa cells undergoing mitosis. (**a**) Tpz-1, (**b**) 1% *v*/*v* DMSO, (**c**) 1 µM Paclitaxel, and (**d**) 5 µg/mL Cytochalasin-D treated cells were stained with α-tubulin-Alexa-488 (microtubules), phalloidin-Alexa-568 (microfilaments), and DAPI (nucleus) prior to examination by confocal microscopy. DMSO was included as vehicle control, and Paclitaxel and Cytochalasin-D as microtubule and microfilament disruptive agents, respectively. Only cells undergoing mitosis were imaged.

For this experiment, Hs27 cells were treated with 10 μM Tpz-1, 5 μg/mL Cytochalasin-D, 1 μM Paclitaxel, or 1% *v*/*v* DMSO for 4 h before fixation and staining. DMSO-treated cells were considered representative of canonical fibroblast morphology, consisting of elongated filaments of both actin and tubulin. Cytochalasin-D and Paclitaxel were used as controls to show microfilament and microtubule organization disruption, respectively. Our analysis revealed a condensed morphology and interruption to microtubule assembly in Hs27 cells treated with Tpz-1 (Figure 6). Similarly, HeLa cells were treated for 24 h with 0.19 μM Tpz-1 or DMSO, Cytochalasin-D, or Paclitaxel controls at the concentrations indicated above. Images were captured at the same stage of mitosis for all treatments, with condensed chromosomes (DAPI; blue) convened at the equatorial plate. Treatment of HeLa with Tpz-1 resulted in multipolar spindle formation (Figure 7). Together these results point to Tpz-1 as a microtubule disrupting agent.

## 4. Discussion

There remains an unmet need for targeted treatments to help patients of all ages with refractory and secondary acute leukemias [23,43,44,45]. This study identified a unique thieno[2,3-c]pyrazole derivative, Tpz-1, that induced cell death of HL-60 acute myeloid leukemia cells at nanomolar concentrations. Apoptosis was validated as the primary mode of Tpz-1-induced cell death in HL-60 cells by detecting Annexin V-FITC bound to exposed phosphatidylserine at the cell surface (Figure 3a). In addition, caspase-8 inactivity (Figure S3) and statistically significant caspase-3/7 activation (Figure 3b), reactive oxygen species accumulation (Figure 3c), and loss of mitochondrial integrity (Figure 3d) were observed by flow cytometry after Tpz-1 exposure and suggest the involvement of the intrinsic apoptotic pathway in compound-induced cell death. Many of these markers for apoptosis were also evident to a comparable degree in the acute lymphocytic leukemia cell line CCRF-CEM (Figures S1 and S2). However, Tpz-1′s effect on the cell cycle of CCRF-CEM remains unclear due to excessive DNA fragmentation at the tested concentrations (Figure S4).

Thienopyrazoles derivatives have well-documented anticancer activity, yet this class of compounds has been mostly ignored. As with their parent moieties, thienopyrazoles have been described in the literature as kinase inhibitors, anti-inflammatory, and receptor modulating agents [10,11,12]. Many previous studies have focused on the activity of thieno[3,2-c]pyrazole derivatives. However, thieno[2,3-c]pyrazole compounds are also emerging as desirable antitumor, antiviral, antimicrobial, and anti-inflammatory agents [14,17,18,46]. Furthermore, highly specific serine/threonine/tyrosine kinase, enzyme, and ligand-gated ion channel antagonist activity have been associated with thieno[2,3-c]pyrazoles [15,47,48,49]. As with similar thienopyrazole derivatives, Tpz-1 disrupted the activity of MAP and Src kinase pathways, resulting in the decreased phosphorylation of p38, CREB, Akt, and STAT3 and hyperphosphorylation of Fgr, Hck, and ERK 1/2.

The MAP kinase (MAPK) pathway plays an essential role in extracellular signal transduction and is often dysregulated in many cancers. Cellular responses associated with MAPK cascades include proliferation, differentiation, and apoptosis [50]. After treatment with Tpz-1, we observed a change in phosphorylation of MAP-associated kinases ERK 1/2, STAT3, CREB, p38, and Akt (Figure 5). ERK 1/2 was one of three kinases hyperphosphorylated after treatment of cells with Tpz-1. ERK is an extracellular signal-regulated kinase whose activation canonically enhances proliferation by controlling cell cycle progression. However, ERK 1/2 activity has alternatively induced G2/M arrest and apoptosis [51,52,53], which we observed with Tpz-1 (Figure 4). Active ERK facilitates the transfer of extracellular signals to the nucleus by phosphorylation of transcription factors such as STAT3 and CREB. STAT3 and CREB are proto-oncogenes often persistently active in cancer, thus making them valuable therapeutic targets [54,55,56]. Despite our observed increase in ERK phosphorylation, STAT3 and CREB phosphorylation was decreased after treatment with Tpz-1. We theorize that this hypophosphorylation of STAT3 and CREB results from Tpz-1’s concomitant antagonism of p38 and Akt, as STAT3 and CREB are also known downstream targets of p38 and Akt signaling pathways [57,58,59,60].

In addition, ERK plays a role in M-phase progression, chromosome alignment, and microtubule assembly [61,62]. Increased ERK phosphorylation by Tpz-1 could thus potentially disrupt microtubules and lead to mitotic spindle dysfunction. Together, this information suggests that ERK activity contributes to the G2/M phase-dependent apoptosis (Figure 4) and microtubule/spindle disruption (Figure 6 and Figure 7) we observed after exposing cells to Tpz-1.

The Src pathway plays an essential role in extracellular signal transduction, and its activation drives tumor progression and metastasis in many cancers. Fgr and Hck are blood cell-specific Src kinases that were hyperphosphorylated in cells exposed to Tpz-1 (Figure 5). Despite its reputation as a proto-oncogene, elevated Hck activity may act as a tumor suppressor in some leukemias, including AML [63,64], which is relevant to our use of HL-60 cells herein. Likewise, Fgr is constitutively active in many cancers but is also known for its ROS-dependent activation and promotion of healthy mitochondrial complex II function [65,66]. Therefore, our observed increase in Fgr phosphorylation could be attributed to ROS accumulation in cells exposed to Tpz-1 (Figure 3c and Figure S1c).

Interestingly, in a previous search for small-molecule mitotic inhibitors that induce apoptosis of multicellular tumor spheroids, Tpz-1 (compound 5248881) was also identified as a lead compound in a screen of over 10,000 molecules from the ChemBridge DIVERset library [19]. The authors revealed that 5248881 induced a similar expression pattern to well-known tubulin inhibitors, inhibited tubulin polymerization in vitro, and caused G2/M cell cycle arrest. Furthermore, this study also determined that 5248881 induced apoptosis by staining the spheroids for caspase-cleaved keratin-18 and active caspase-3 [19].

Our results did not show arrest but have shown that Tpz-1 interferes with the cell cycle (Figure 4), causes cell death and a concurrent loss of cells in the G2/M phase in the HL-60 cell line (Figure 4a,c), and damages the mitotic spindle of dividing cells (Figure 7). Furthermore, another recently published study revealed that Tpz-1 (compound MTPC) could target tumors in mice by encapsulating the compound in gold nanorod-loaded PLGA-PEG nanoparticles [20]. Together, these previous publications and the current study support the continued development of Tpz-1 as a chemotherapeutic candidate.

In conclusion, Tpz-1 is a potent and cytotoxic thieno[2,3-c]pyrazole derivative with potent and specific anticancer activity in vitro. Despite compelling evidence of the diverse biological activity of thienopyrazoles, these findings have brought to light a scarcity of publications focused on this unique scaffold. While the contribution of the thienopyrazole moiety to Tpz-1’s biological activity remains unknown, our data parallels previously studied thieno[2,3-c]pyrazoles. Research to further elucidate Tpz-1’s mechanism of action is ongoing. Until then, we hope that this work inspires our colleagues to consider thienopyrazole-based compounds in small-molecule anticancer drug formulations.

## Figures and Tables

**Table 1:** Cytotoxic concentration 50% (CC_50_) of Tpz-1 in a panel of 17 cancer and 1 non-cancerous cell line at 24 h (top), 48 h, and 72 h (bottom) of exposure.

Hematological Cancer Cell Lines		
Cell Line	Origin	CC_50_ (µM ± St. Dev) ^a^
CCRF-CEM	T-cell leukemia	0.74 ± 0.18 (24 h)
		0.25 ± 0.04 (48 h)
		0.19 ± 0.01 (72 h)
HL-60	Acute myeloid leukemia	0.95 ± 0.03 (24 h)
		0.63 ± 0.03 (48 h)
		0.27 ± 0.03 (72 h)
Ramos	Burkitt’s lymphoma	0.30 ± 0.01 (48 h)
Jurkat	T-cell leukemia	0.41 ± 0.004 (48 h)
MOLT-3	T-cell leukemia	2.10 ± 0.14 (48 h)
NALM6	B-cell precursor leukemia	0.24 ± 0.002 (48 h)
MM.1S	Multiple myeloma	0.82 ± 0.08 (48 h)
MM.1R	Dexamethasone-resistant multiple myeloma	0.78 ± 0.02 (48 h)
U266	Multiple myeloma	0.93 ± 0.13 (48 h)
RPMI 8226	Multiple myeloma	0.33 ± 0.01 (48 h)
**Non-Tumorigenic Cell Lines**		
Hs27 *	Foreskin fibroblast	6.11 ± 0.03 (48 h)
		5.72 ± 2.29 (72 h)
**Solid Tumor Cell Lines**		
MDA-MB-231	Triple-negative breast adenocarcinoma	1.94 ± 0.19 (72 h)
MDA-MB-468	Triple-negative breast adenocarcinoma	0.80 ± 0.07 (72 h)
MCF7	ER+ breast adenocarcinoma	2.99 ± 0.67 (72 h)
COLO 205	Colorectal adenocarcinoma	0.93 ± 0.03 (72 h)
HT-29	Colorectal adenocarcinoma	0.91 ± 0.06 (72 h)
HeLa	Cervical adenocarcinoma	0.68 ± 0.04 (72 h)
SH-SY5Y	Neuroblastoma	0.21 ± 0.01 (72 h)

^a^ The CC_50_ refers to the concentration at which 50% of the cell population dies after 24 h, 48 h, or 72 h Tpz-1 exposure; ± values denote standard deviations; * Indicate non-tumorigenic cell lines.

**Table 2:** Selective cytotoxicity index (SCI) values of Tpz-1 in 17 cancer cell lines at 48 h (top) and 72 h (bottom) of exposure.

Cell Line	SCI 48 h
CCRF-CEM	24.44
HL-60	9.70
Ramos	20.37
Jurkat	14.90
MOLT-3	2.91
NALM6	25.46
MM.1S	7.45
MM.1R	7.83
U266	6.57
RPMI 8226	18.52
**Cell Line**	**SCI 72 h**
CCRF-CEM	30.11
HL-60	21.19
MDA-MB-231	2.95
MDA-MB-468	7.15
MCF7	1.91
COLO 205	6.15
HT-29	6.29
HeLa	8.41
SH-SY5Y	27.24

## Data Availability

The data presented in this study are available on request from the corresponding author. The data are not publicly available due to file size and quantity.

## Supplementary Materials

The following supporting information can be downloaded at: https://www.mdpi.com/article/10.3390/biology11060930/s1, Figure S1: Tpz-1-induced cell death is mediated by apoptosis in CCRF-CEM cells; Figure S2: Caspase-3/7 activity is reduced after 8 h exposure to Tpz-1 in CCRF-CEM cells; Figure S3: Caspase-8 is inactive after 4 h in Tpz-1-treated HL-60 cells; Figure S4: Tpz-1 causes DNA fragmentation that interferes with evaluation of the CCRF-CEM cell cycle.

## References

[1] Bedard P.L., Hyman D.M., Davids M.S., Siu L.L. (2020). Small molecules, big impact: 20 years of targeted therapy in oncology. Lancet.

[2] Lee Y.T., Tan Y.J., Oon C.E. (2018). Molecular targeted therapy: Treating cancer with specificity. Eur. J. Pharmacol..

[3] Zhong L., Li Y., Xiong L., Wang W., Wu M., Yuan T., Yang W., Tian C., Miao Z., Wang T. (2021). Small molecules in targeted cancer therapy: Advances, challenges, and future perspectives. Signal Transduct. Target. Ther..

[4] Karrouchi K., Radi S., Ramli Y., Taoufik J., Mabkhot Y.N., Al-Aizari F.A., Ansar M. (2018). Synthesis and pharmacological activities of pyrazole derivatives: A review. Molecules.

[5] Shah R., Verma P.K. (2018). Therapeutic importance of synthetic thiophene. Chem. Cent. J..

[6] El-Gamal M.I., Zaraei S.-O., Madkour M.M., Anbar H.S. (2022). Evaluation of substituted pyrazole-based kinase inhibitors in one decade (2011–2020): Current status and future prospects. Molecules.

[7] Gutierrez D.A., Contreras L., Villanueva P.J., Borrego E.A., Morán-Santibañez K., Hess J.D., DeJesus R., Larragoity M., Betancourt A.P., Mohl J.E. (2022). Identification of a potent cytotoxic pyrazole with anti-breast cancer activity that alters multiple pathways. Cells.

[8] Ashourpour M., Mostafavi Hosseini F., Amini M., Saeedian Moghadam E., Kazerouni F., Arman S.Y., Shahsavari Z. (2021). Pyrazole derivatives induce apoptosis via ROS generation in the triple negative breast cancer cells, MDA-MB-468. Asian Pac. J. Cancer Prev..

[9] Archna, Pathania S., Chawla P.A. (2020). Thiophene-based derivatives as anticancer agents: An overview on decade’s work. Bioorg. Chem..

[10] El-Borai M.A., Rizk H.F., Ibrahim S.A., Fares A.K., El-Tahawy M.M.T., Beltagy D.M. (2021). Assessment of anti-hemolytic, cytotoxicity, antioxidant activities and molecular docking study based on thienopyrazole scaffold as pharmacophore. J. Mol. Struct..

[11] Raffa D., Maggio B., Raimondi M.V., Cascioferro S., Plescia F., Cancemi G., Daidone G. (2015). Recent advanced in bioactive systems containing pyrazole fused with a five membered heterocycle. Eur. J. Med. Chem..

[12] Zaki R.M., Saber A.F., El-Dean A.M.K., Radwan S.M. (2020). A concise review on synthesis, reactions and biological importance of thienopyrazoles. ARKIVOC.

[13] Goto M., Murakawa M., Kadoshima-Yamaoka K., Tanaka Y., Inoue H., Murafuji H., Hayashi Y., Miura K., Nakatsuka T., Nagahira K. (2009). Phosphodiesterase 7A inhibitor ASB16165 suppresses proliferation and cytokine production of NKT cells. Cell. Immunol..

[14] Goto M., Kadoshima-Yamaoka K., Murakawa M., Yoshioka R., Tanaka Y., Inoue H., Murafuji H., Kanki S., Hayashi Y., Nagahira K. (2010). Phosphodiesterase 7A inhibitor ASB16165 impairs proliferation of keratinocytes in vitro and in vivo. Eur. J. Pharmacol..

[15] Brotherton-Pleiss C.E., Dillon M.P., Ford A.P.D.W., Gever J.R., Carter D.S., Gleason S.K., Lin C.J., Moore A.G., Thompson A.W., Villa M. (2010). Discovery and optimization of RO-85, a novel drug-like, potent, and selective P2X3 receptor antagonist. Bioorg. Med. Chem. Lett..

[16] Fancelli D., Isacchi A., Modugno M., Moll J., Rusconi L., Soncini C., Lupi R. (2010). Use of a Kinase Inhibitor for the Treatment of Particular Resistant Tumors. U.S. Patent.

[17] Fancelli D., Moll J., Pulici M., Quartieri F., Bandiera T. (2011). 1H-Thieno[2,3-c]Pyrazole Compounds Useful as Kinase Inhibitors. U.S. Patent.

[18] Barberis C., Carry J.-C., Doerflinger G., Barbalat-Damour D., Clerc F., Minoux H. (2009). Hydrazinocarbonyl-Thieno[2,3-C]Pyrazoles, Process for Preparing Them, Compositions Containing Them and Use Thereof. U.S. Patent.

[19] Fayad W., Rickardson L., Haglund C., Olofsson M.H., D’Arcy P., Larsson R., Linder S., Fryknäs M. (2011). Identification of agents that induce apoptosis of multicellular tumour spheroids: Enrichment for mitotic inhibitors with hydrophobic properties. Chem. Biol. Drug Des..

[20] Darwish W.M.A., Bayoumi N.A. (2020). Gold Nanorod-Loaded (PLGA-PEG) Nanocapsules as near-infrared controlled release model of anticancer therapeutics. Lasers Med. Sci..

[21] Siegel R.L., Miller K.D., Fuchs H.E., Jemal A. (2022). Cancer statistics, 2022. CA Cancer J. Clin..

[22] Miller K.D., Fidler-Benaoudia M., Keegan T.H., Hipp H.S., Jemal A., Siegel R.L. (2020). Cancer statistics for adolescents and young adults, 2020. CA Cancer J. Clin..

[23] Hijiya N., Ness K.K., Ribeiro R.C., Hudson M.M. (2009). Acute leukemia as a secondary malignancy in children and adolescents: Current findings and issues. Cancer.

[24] Leone G., Mele L., Pulsoni A., Equitani F., Pagano L. (1999). The incidence of secondary leukemias. Haematologica.

[25] Bispo J.A.B., Pinheiro P.S., Kobetz E.K. (2020). Epidemiology and etiology of leukemia and lymphoma. Cold Spring Harb. Perspect. Med..

[26] Shallis R.M., Wang R., Davidoff A., Ma X., Zeidan A.M. (2019). Epidemiology of acute myeloid leukemia: Recent progress and enduring challenges. Blood Rev..

[27] Miller K.D., Nogueira L., Mariotto A.B., Rowland J.H., Yabroff K.R., Alfano C.M., Jemal A., Kramer J.L., Siegel R.L. (2019). Cancer treatment and survivorship statistics, 2019. CA Cancer J. Clin..

[28] Ruiz-Medina B.E., Lerma D., Hwang M., Ross J.A., Skouta R., Aguilera R.J., Kirken R.A., Varela-Ramirez A., Robles-Escajeda E. (2019). Green barley mitigates cytotoxicity in human lymphocytes undergoing aggressive oxidative stress, via activation of both the Lyn/PI3K/Akt and MAPK/ERK pathways. Sci. Rep..

[29] Lema C., Varela-Ramirez A., Aguilera R.J. (2011). Differential nuclear staining assay for high-throughput screening to identify cytotoxic compounds. Curr. Cell. Biochem..

[30] Cook J.D. Linear Interpolation Calculator. https://www.johndcook.com/interpolator.html.

[31] Nakamura-Bencomo S., Gutierrez D.A., Robles-Escajeda E., Iglesias-Figueroa B., Siqueiros-Cendón T.S., Espinoza-Sánchez E.A., Arévalo-Gallegos S., Aguilera R.J., Rascón-Cruz Q., Varela-Ramirez A. (2021). Recombinant human lactoferrin carrying humanized glycosylation exhibits antileukemia selective cytotoxicity, microfilament disruption, cell cycle arrest, and apoptosis activities. Investig. New Drugs.

[32] Robles-Escajeda E., Das U., Ortega N.M., Parra K., Francia G., Dimmock J.R., Varela-Ramirez A., Aguilera R.J. (2016). A novel curcumin-like dienone induces apoptosis in triple-negative breast cancer cells. Cell. Oncol..

[33] Pistritto G., Trisciuoglio D., Ceci C., Garufi A., D’Orazi G. (2016). Apoptosis as anticancer mechanism: Function and dysfunction of its modulators and targeted therapeutic strategies. Aging.

[34] Robles-Escajeda E., Lerma D., Nyakeriga A.M., Ross J.A., Kirken R.A., Aguilera R.J., Varela-Ramirez A. (2013). Searching in mother nature for anti-cancer activity: Anti-proliferative and pro-apoptotic effect elicited by green barley on leukemia/lymphoma cells. PLoS ONE.

[35] Tummers B., Green D.R. (2017). Caspase-8: Regulating life and death. Immunol. Rev..

[36] Sinha K., Das J., Pal P.B., Sil P.C. (2013). Oxidative Stress: The mitochondria-dependent and mitochondria-independent pathways of apoptosis. Arch. Toxicol..

[37] Gutierrez D.A., DeJesus R.E., Contreras L., Rodriguez-Palomares I.A., Villanueva P.J., Balderrama K.S., Monterroza L., Larragoity M., Varela-Ramirez A., Aguilera R.J. (2019). A new pyridazinone exhibits potent cytotoxicity on human cancer cells via apoptosis and poly-ubiquitinated protein accumulation. Cell Biol. Toxicol..

[38] Kaisar M.A., Sivandzade F., Bhalerao A., Cucullo L. (2018). Conventional and electronic cigarettes dysregulate the expression of iron transporters and detoxifying enzymes at the brain vascular endothelium: In Vivo evidence of a gender-specific cellular response to chronic cigarette smoke exposure. Neurosci. Lett..

[39] Sivandzade F., Bhalerao A., Cucullo L. (2019). Analysis of the mitochondrial membrane potential using the cationic JC-1 dye as a sensitive fluorescent probe. Bio Protoc..

[40] Martin S.J., Reutelingsperger C.P., McGahon A.J., Rader J.A., van Schie R.C., LaFace D.M., Green D.R. (1995). Early redistribution of plasma membrane phosphatidylserine is a general feature of apoptosis regardless of the initiating stimulus: Inhibition by overexpression of Bcl-2 and Abl. J. Exp. Med..

[41] Redza-Dutordoir M., Averill-Bates D.A. (2016). Activation of apoptosis signalling pathways by reactive oxygen species. Biochim. Biophys. Acta.

[42] Tait S.W.G., Green D.R. (2010). Mitochondria and cell death: Outer membrane permeabilization and beyond. Nat. Rev. Mol. Cell Biol..

[43] Liu H. (2021). Emerging agents and regimens for AML. J. Hematol. Oncol..

[44] Al-Hussaini M., DiPersio J.F. (2014). Small molecule inhibitors in acute myeloid leukemia: From the bench to the clinic. Expert Rev. Hematol..

[45] Malard F., Mohty M. (2020). Acute lymphoblastic leukaemia. Lancet.

[46] El-Dean A.M.K., Zaki R.M., Abdulrazzaq A.Y. (2015). A convenient synthesis and biological activity of novel Thieno[2,3-c]Pyrazole compounds as antimicrobial and anti-inflammatory agents. Russ. J. Bioorg. Chem..

[47] McLean L.R., Zhang Y., Zaidi N., Bi X., Wang R., Dharanipragada R., Jurcak J.G., Gillespy T.A., Zhao Z., Musick K.Y. (2012). X-Ray Crystallographic Structure-based design of selective thienopyrazole inhibitors for interleukin-2-inducible tyrosine kinase. Bioorg. Med. Chem. Lett..

[48] Akritopoulou-Zanze I., Hajduk P.J. (2009). Kinase-targeted libraries: The design and synthesis of novel, potent, and selective kinase inhibitors. Drug Discov. Today.

[49] Akritopoulou-Zanze I., Darczak D., Sarris K., Phelan K.M., Huth J.R., Song D., Johnson E.F., Jia Y., Djuric S.W. (2006). Scaffold oriented synthesis. part 1: Design, preparation, and biological evaluation of thienopyrazoles as kinase inhibitors. Bioorg. Med. Chem. Lett..

[50] Dhillon A.S., Hagan S., Rath O., Kolch W. (2007). MAP kinase signalling pathways in cancer. Oncogene.

[51] Yan Y., Black C.P., Cowan K.H. (2007). Irradiation-induced G2/M checkpoint response requires ERK1/2 activation. Oncogene.

[52] Lv C., Hong Y., Miao L., Li C., Xu G., Wei S., Wang B., Huang C., Jiao B. (2013). Wentilactone A as a novel potential antitumor agent induces apoptosis and G2/M arrest of human lung carcinoma cells, and is mediated by HRas-GTP accumulation to excessively activate the Ras/Raf/ERK/P53-P21 pathway. Cell Death Dis..

[53] Wang X., Martindale J.L., Holbrook N.J. (2000). Requirement for ERK activation in cisplatin-induced apoptosis. J. Biol. Chem..

[54] Kamran M.Z., Patil P., Gude R.P. (2013). Role of STAT3 in cancer metastasis and translational advances. Biomed. Res. Int..

[55] Xiao X., Li B.X., Mitton B., Ikeda A., Sakamoto K.M. (2010). Targeting CREB for cancer therapy: Friend or foe. Curr. Cancer Drug Targets.

[56] Huynh J., Chand A., Gough D., Ernst M. (2019). Therapeutically exploiting STAT3 activity in cancer—Using tissue repair as a road map. Nat. Rev. Cancer.

[57] Riebe C., Pries R., Schroeder K.N., Wollenberg B. (2011). Phosphorylation of STAT3 in head and neck cancer requires P38 MAPKinase, whereas phosphorylation of STAT1 occurs via a different signaling pathway. Anticancer. Res..

[58] Du K., Montminy M. (1998). CREB Is a regulatory target for the protein kinase Akt/PKB. J. Biol. Chem..

[59] Liu J., Chen B., Lu Y., Guan Y., Chen F. (2012). JNK-Dependent Stat3 phosphorylation contributes to Akt activation in response to arsenic exposure. Toxicol. Sci..

[60] Delghandi M.P., Johannessen M., Moens U. (2005). The CAMP signalling pathway activates CREB through PKA, P38 and MSK1 in NIH 3T3 cells. Cell. Signal..

[61] Iwamoto E., Ueta N., Matsui Y., Kamijo K., Kuga T., Saito Y., Yamaguchi N., Nakayama Y. (2016). ERK plays a role in chromosome alignment and participates in M-phase progression. J. Cell. Biochem..

[62] Verlhac M.H., de Pennart H., Maro B., Cobb M.H., Clarke H.J. (1993). MAP kinase becomes stably activated at metaphase and is associated with microtubule-organizing centers during meiotic maturation of mouse oocytes. Dev. Biol..

[63] Zou D., Yang X., Tan Y., Wang P., Zhu X., Yang W., Jia X., Zhang J., Wang K. (2012). Regulation of the hematopoietic cell kinase (HCK) by PML/RARα and PU.1 in acute promyelocytic leukemia. Leuk. Res..

[64] Shivakrupa R., Radha V., Sudhakar C., Swarup G. (2003). Physical and functional interaction between Hck tyrosine kinase and guanine nucleotide exchange factor C3G results in apoptosis, which is independent of C3G catalytic domain. J. Biol. Chem..

[65] Tibaldi E., Brunati A.M., Massimino M.L., Stringaro A., Colone M., Agostinelli E., Arancia G., Toninello A. (2008). Src-Tyrosine kinases are major agents in mitochondrial tyrosine phosphorylation. J. Cell. Biochem..

[66] Acín-Pérez R., Carrascoso I., Baixauli F., Roche-Molina M., Latorre-Pellicer A., Fernández-Silva P., Mittelbrunn M., Sanchez-Madrid F., Pérez-Martos A., Lowell C.A. (2014). ROS-triggered phosphorylation of complex II by Fgr kinase regulates cellular adaptation to fuel use. Cell Metab..

