# Supplementary material for: Identification of a Unique Cytotoxic Thieno[2,3-c]Pyrazole Derivative with Potent and Selective Anticancer Effects In Vitro
Source: Biology (Basel). 2022 Jun 18;11(6):930. doi: 10.3390/biology11060930 (PMC9219615; doi:10.3390/biology11060930)
Supplement: Supplementary file 1 [file biology-11-00930-s001.zip › biology-1741470-supplementary.pdf]

Supplemental Figures

Figure S1a-d

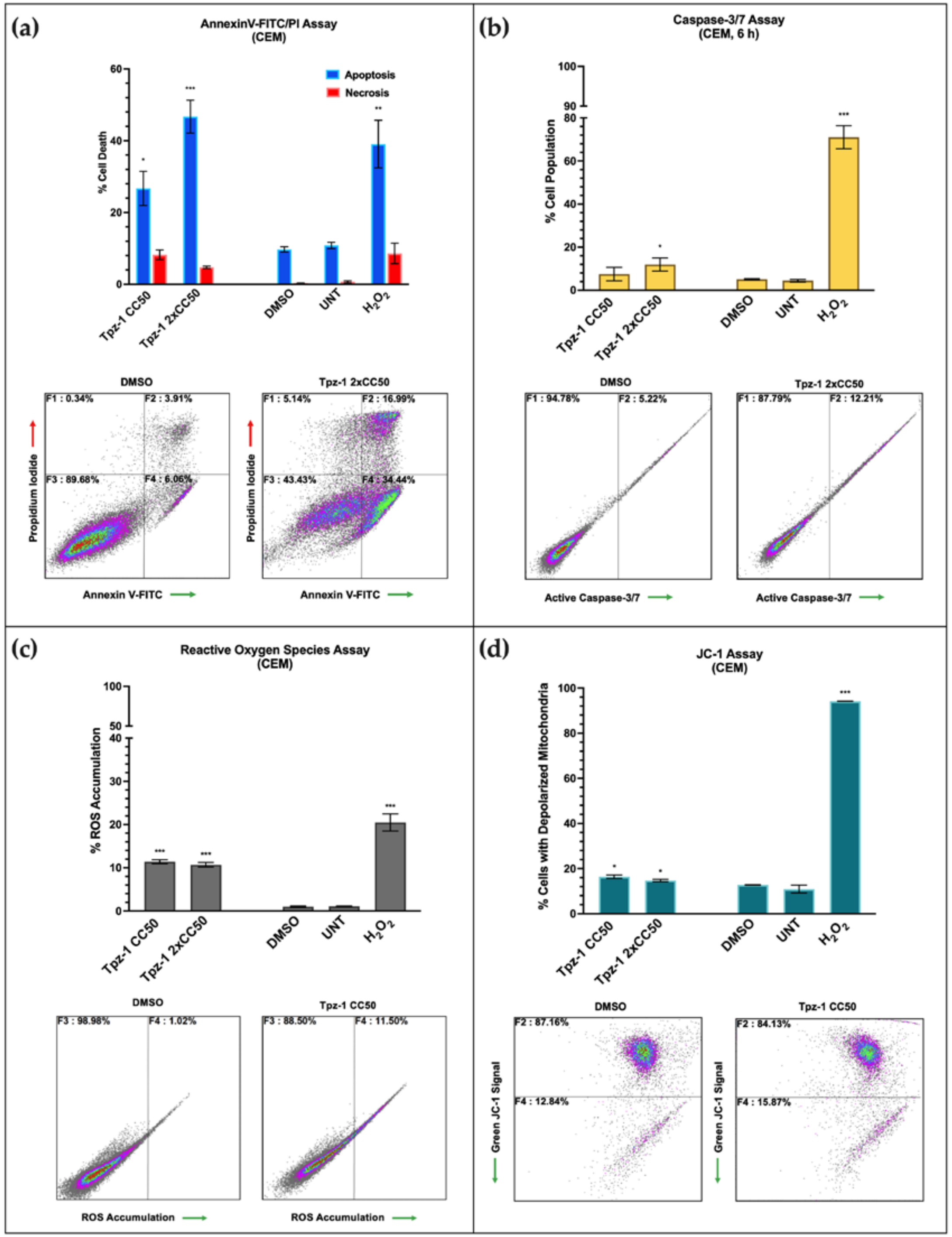

(a)
AnnexinV-FITC/PI Assay
(CEM)
Apoptosis
Necrosis
% Cell Death
Tpz-1 CC50
Tpz-1 2xCC50
DMSO
UNT
H2O2
DMSO
Tpz-1 2xCC50
F1 : 0.34%
F2 : 3.91%
F3 : 89.68%
F4 : 6.06%
F1 : 5.14%
F2 : 16.99%
F3 : 43.43%
F4 : 34.44%
Propidium Iodide
Annexin V-FITC
(b)
Caspase-3/7 Assay
(CEM, 6 h)
% Cell Population
Tpz-1 CC50
Tpz-1 2xCC50
DMSO
UNT
H2O2
DMSO
Tpz-1 2xCC50
F1 : 94.78%
F2 : 5.22%
F1 : 87.79%
F2 : 12.21%
Active Caspase-3/7
(c)
Reactive Oxygen Species Assay
(CEM)
% ROS Accumulation
Tpz-1 CC50
Tpz-1 2xCC50
DMSO
UNT
H2O2
DMSO
Tpz-1 CC50
F3 : 98.98%
F4 : 1.02%
F3 : 88.50%
F4 : 11.50%
ROS Accumulation
(d)
JC-1 Assay
(CEM)
% Cells with Depolarized Mitochondria
Tpz-1 CC50
Tpz-1 2xCC50
DMSO
UNT
H2O2
DMSO
Tpz-1 CC50
F2 : 87.16%
F4 : 12.84%
F2 : 84.13%
F4 : 15.87%
Green JC-1 Signal

Figure S1a

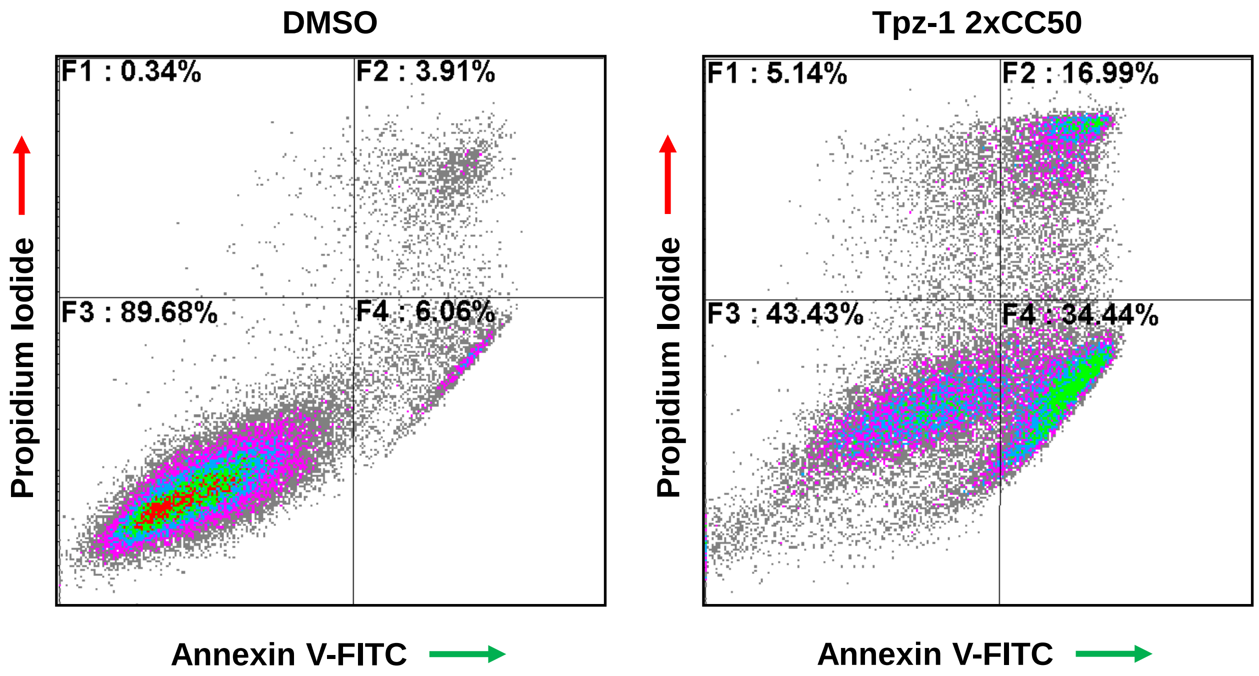

DMSO
F1 : 0.34%
F2 : 3.91%
F3 : 89.68%
F4 : 6.06%
Propidium Iodide
Annexin V-FITC
Tpz-1 2xCC50
F1 : 5.14%
F2 : 16.99%
F3 : 43.43%
F4 : 34.44%
Propidium Iodide
Annexin V-FITC

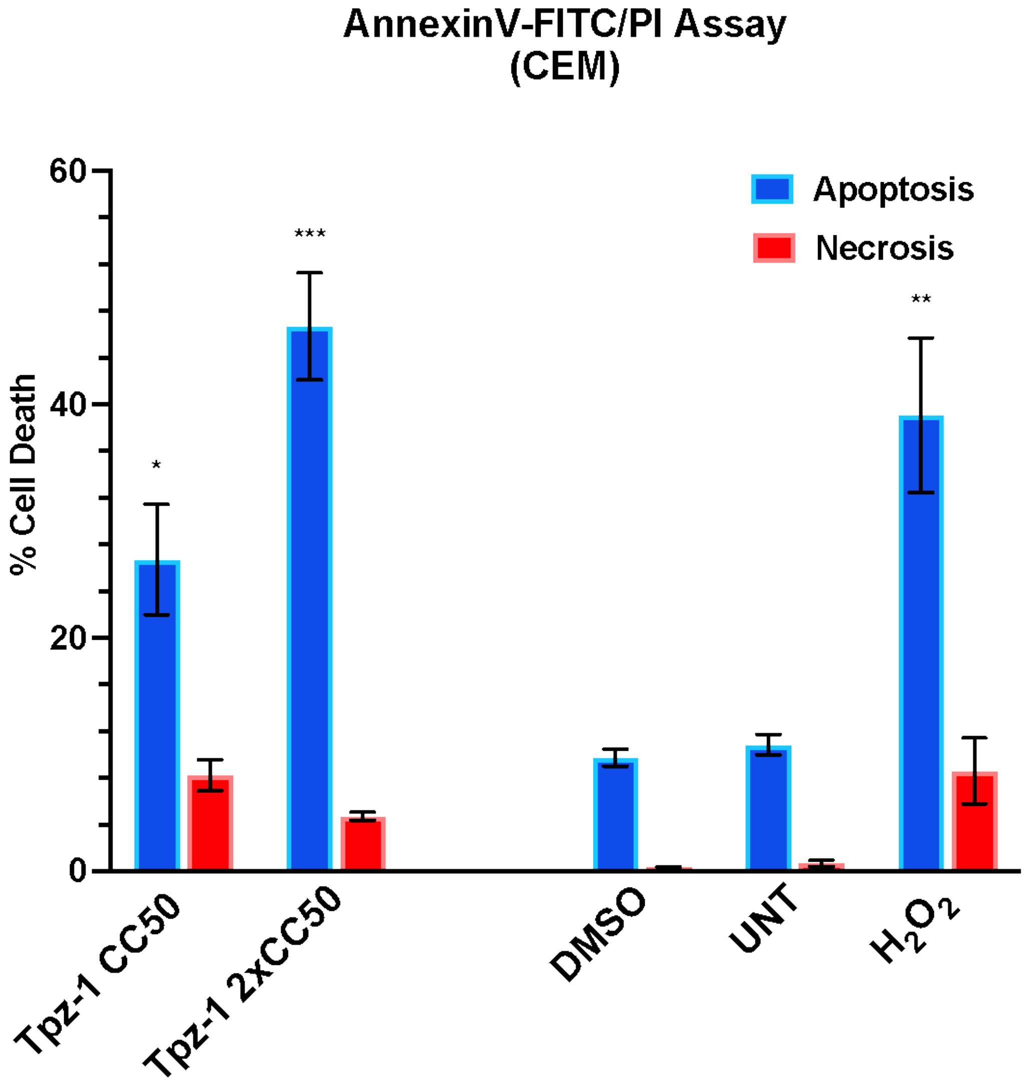

AnnexinV-FITC/PI Assay
(CEM)
% Cell Death
60
40
20
0
Apoptosis
Necrosis
*
***
**
Tpz-1 CC50
Tpz-1 2xCC50
DMSO
UNT
H_2O_2

Figure S1b

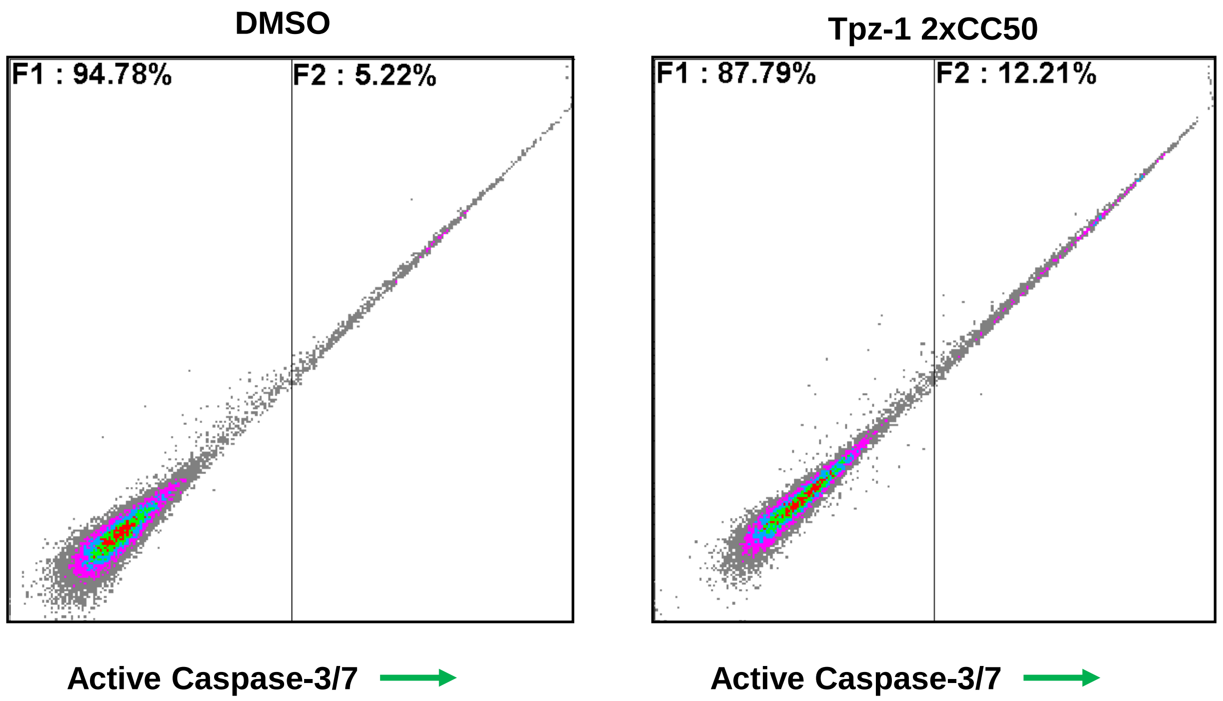
DMSO
F1 : 94.78%
F2 : 5.22%
Active Caspase-3/7
Tpz-1 2xCC50
F1 : 87.79%
F2 : 12.21%
Active Caspase-3/7

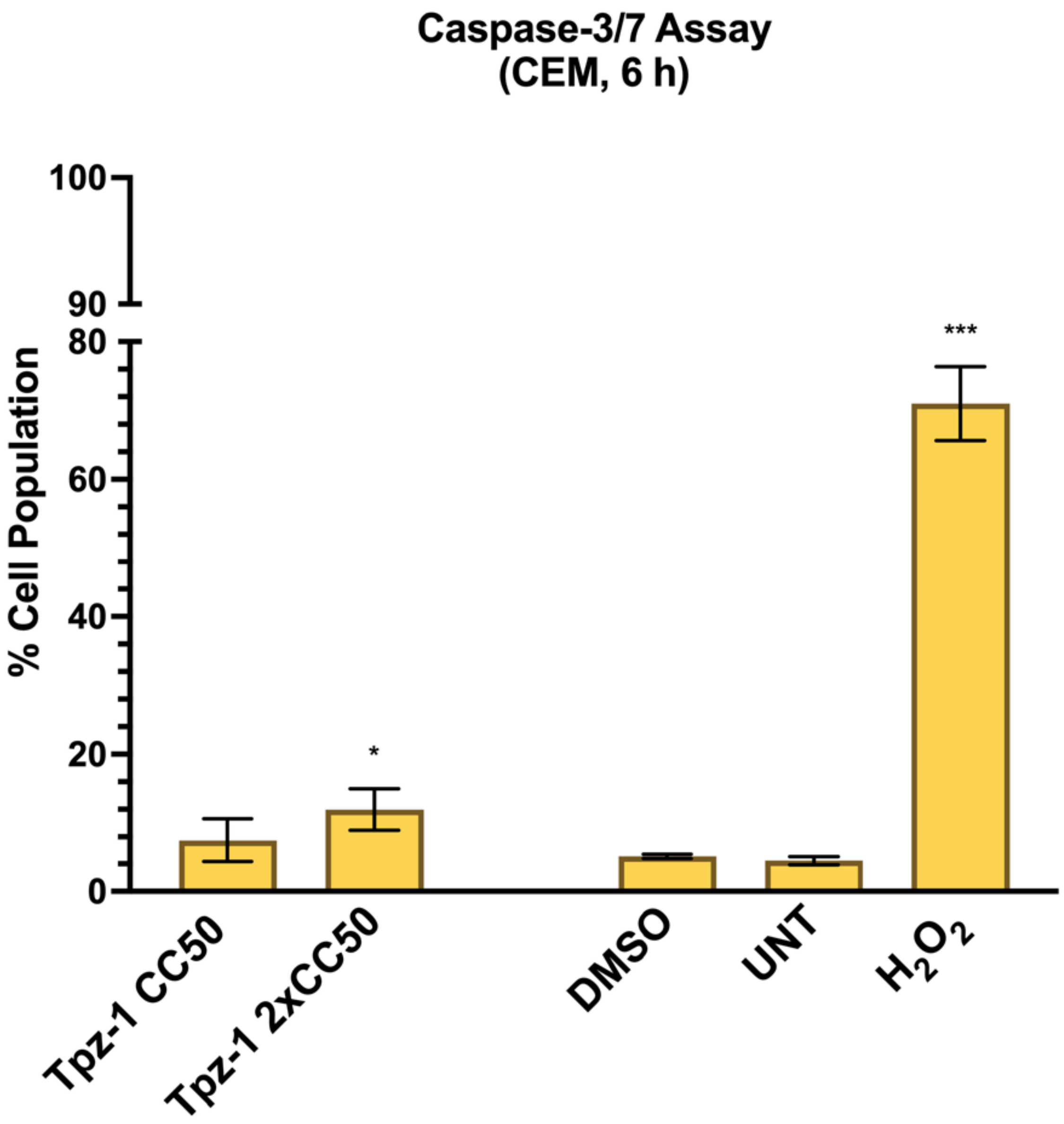
Caspase-3/7 Assay
(CEM, 6 h)
% Cell Population
100
90
80
60
40
20
0
*
***
Tpz-1 CC50
Tpz-1 2xCC50
DMSO
UNT
H2O2

Figure S1c

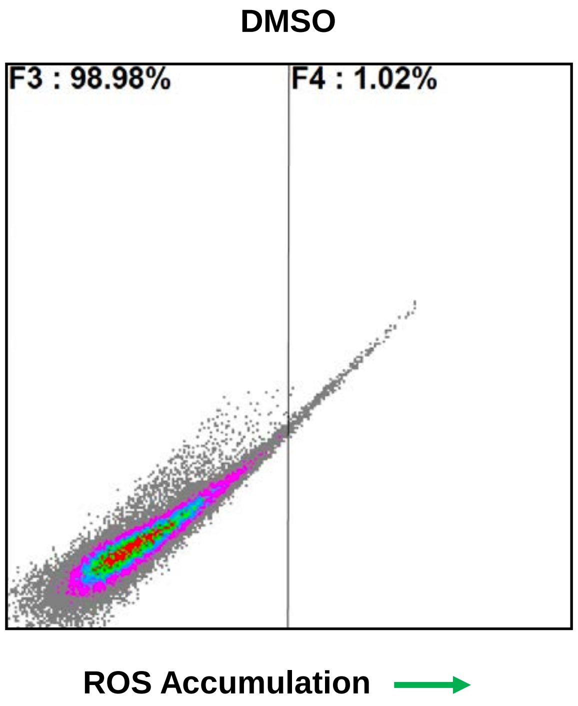
DMSO
F3 : 98.98%
F4 : 1.02%
ROS Accumulation

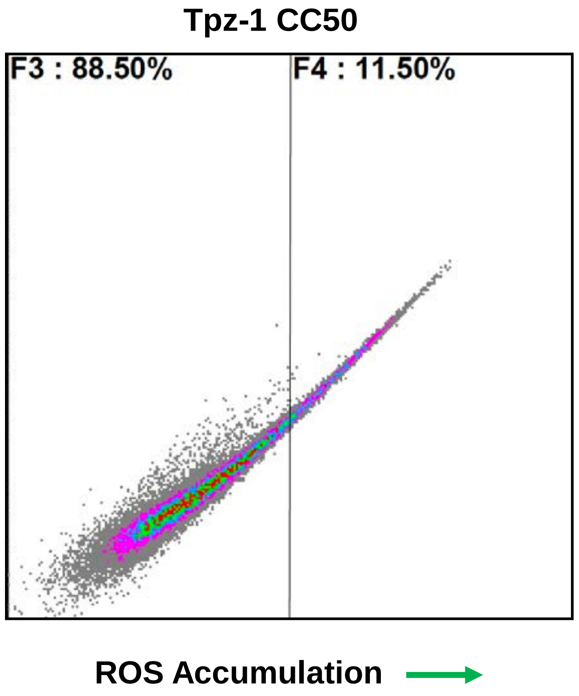
Tpz-1 CC50
F3 : 88.50%
F4 : 11.50%
ROS Accumulation

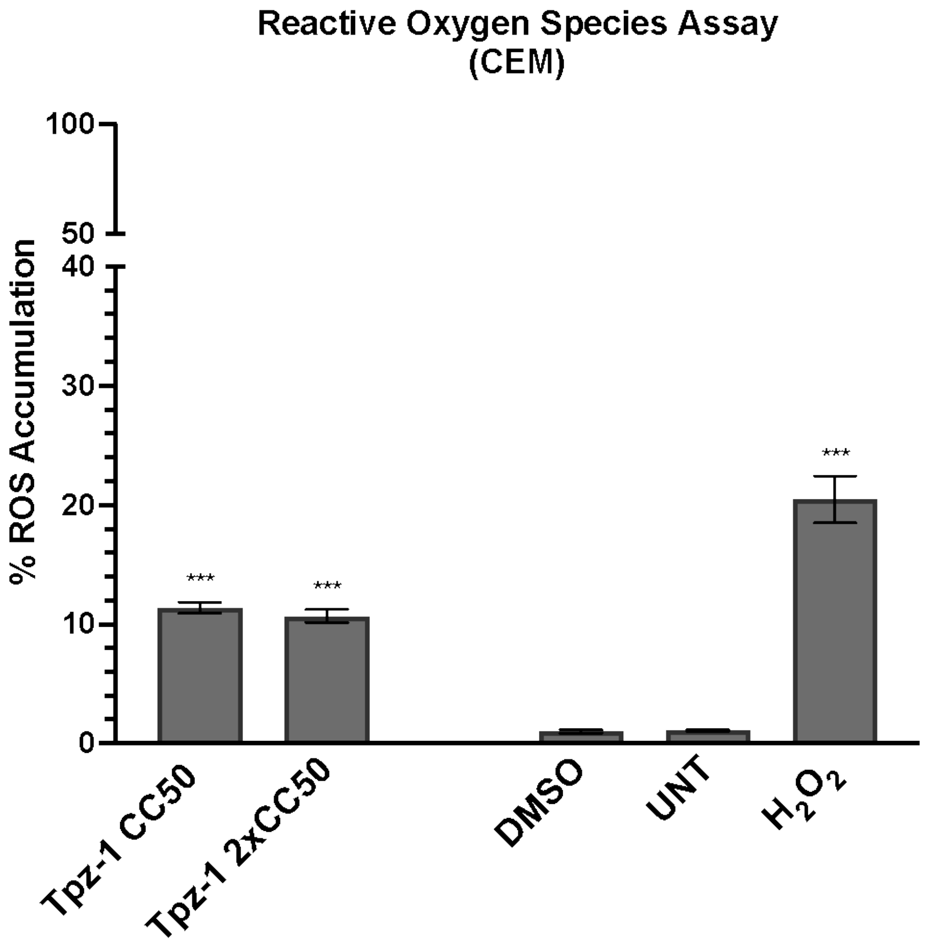
Reactive Oxygen Species Assay
(CEM)
% ROS Accumulation
100
50
40
30
20
10
0
***
***
***
Tpz-1 CC50
Tpz-1 2xCC50
DMSO
UNT
H2O2

Figure S1d

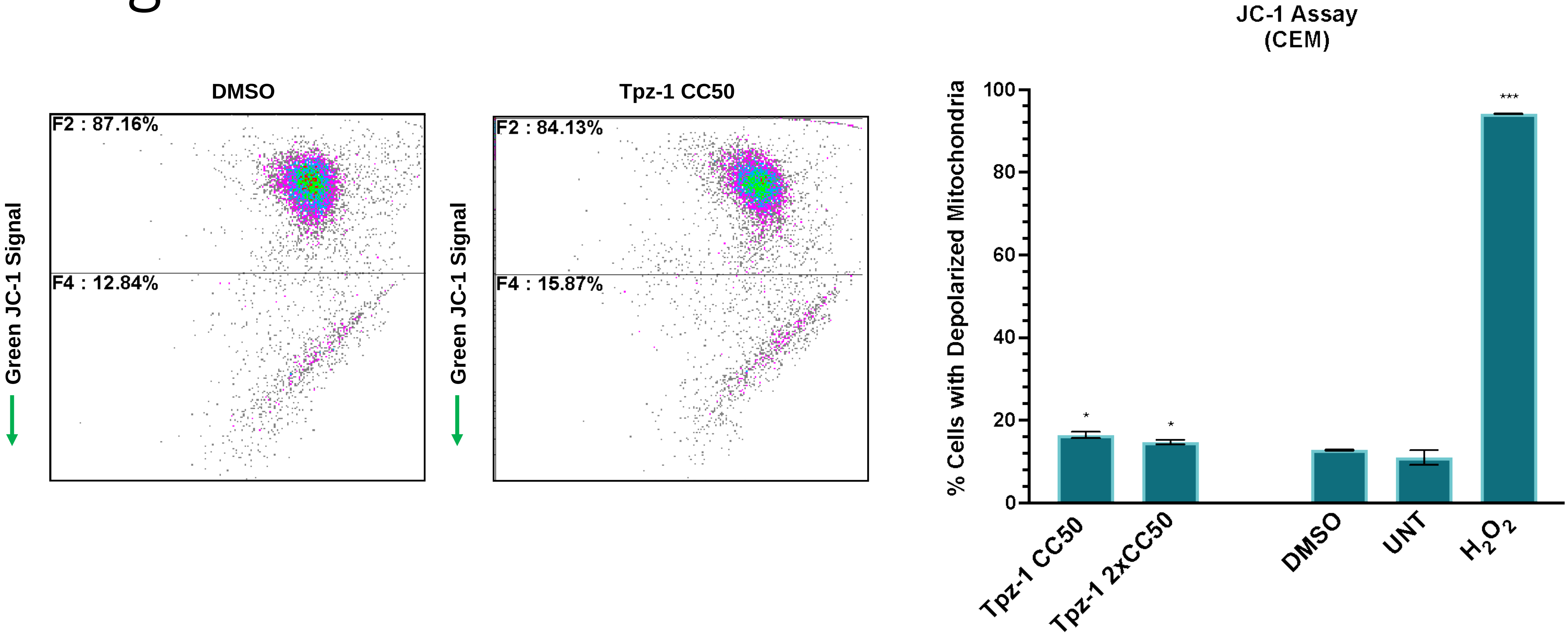
DMSO
F2 : 87.16%
F4 : 12.84%
Green JC-1 Signal
Tpz-1 CC50
F2 : 84.13%
F4 : 15.87%
Green JC-1 Signal
JC-1 Assay
(CEM)
% Cells with Depolarized Mitochondria
100
80
60
40
20
0
*
*
***
Tpz-1 CC50
Tpz-1 2xCC50
DMSO
UNT
H_2O_2

Figure S2

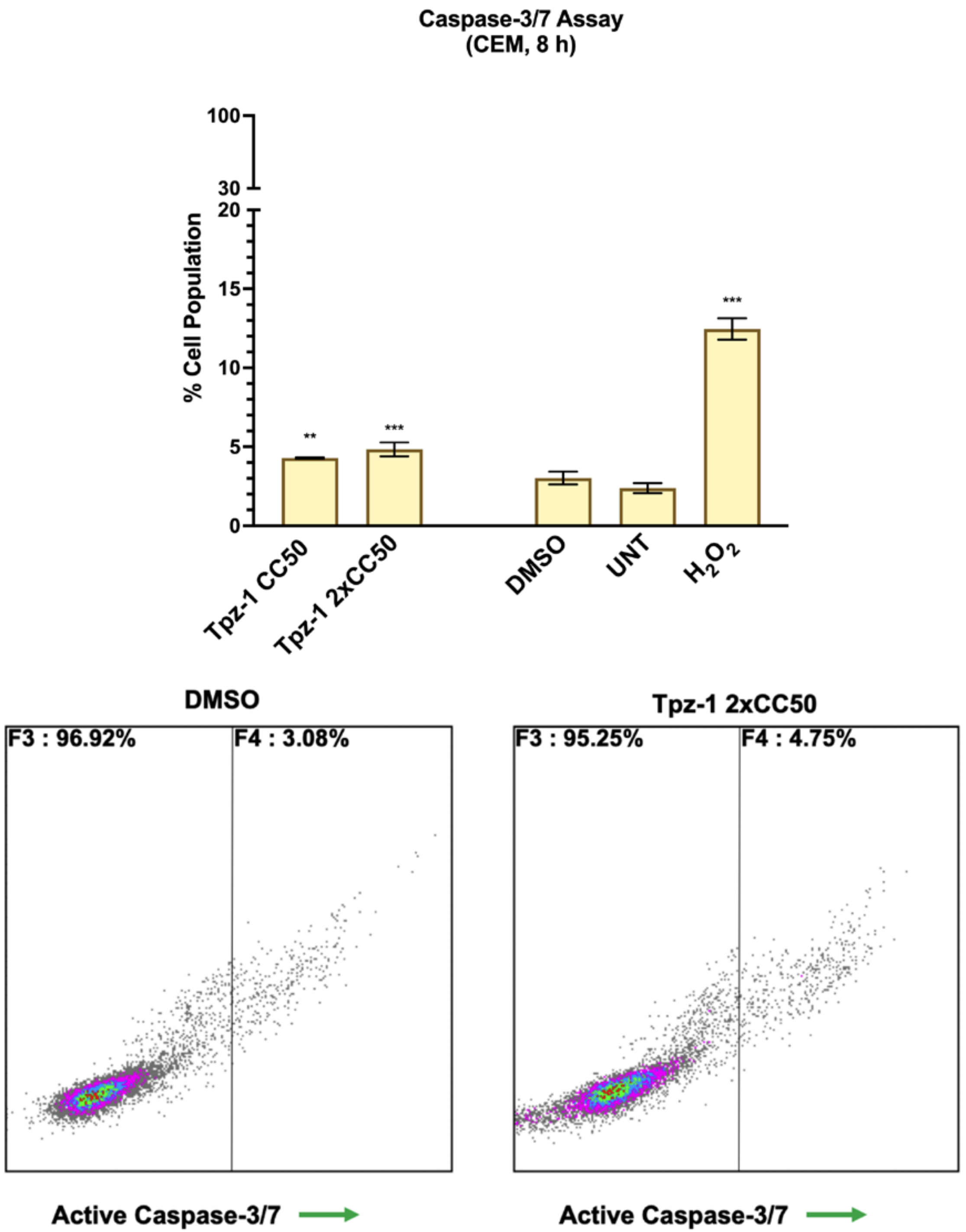
Caspase-3/7 Assay
(CEM, 8 h)
% Cell Population
100
30
20
15
10
5
0
**
***
***
Tpz-1 CC50
Tpz-1 2xCC50
DMSO
UNT
H_2O_2
DMSO
F3 : 96.92%
F4 : 3.08%
Active Caspase-3/7
Tpz-1 2xCC50
F3 : 95.25%
F4 : 4.75%
Active Caspase-3/7

Figure S3

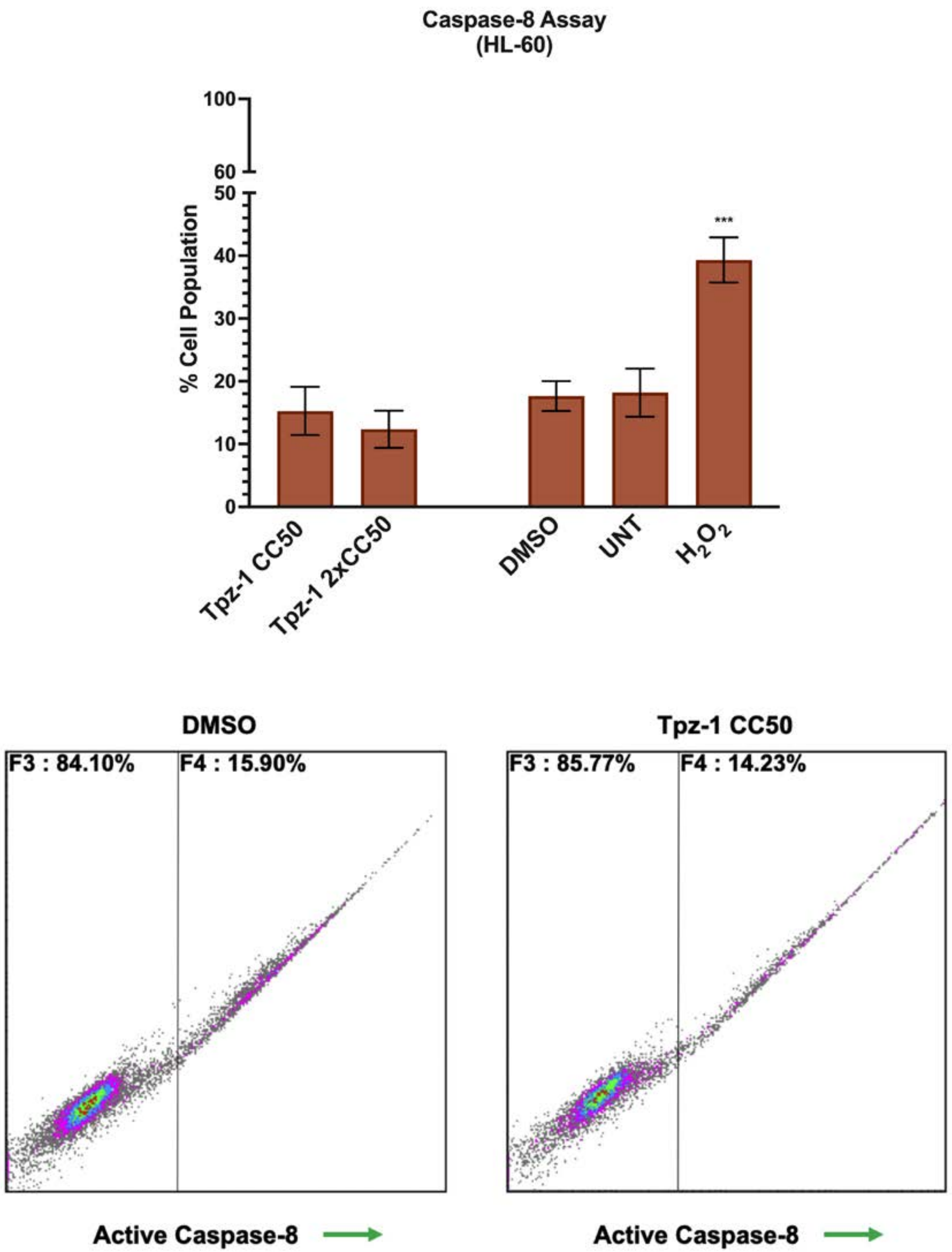
Caspase-8 Assay
(HL-60)
% Cell Population
100
60
50
40
30
20
10
0
***
Tpz-1 CC50
Tpz-1 2xCC50
DMSO
UNT
H2O2
DMSO
F3 : 84.10%
F4 : 15.90%
Active Caspase-8
Tpz-1 CC50
F3 : 85.77%
F4 : 14.23%
Active Caspase-8

Figure S4a-d

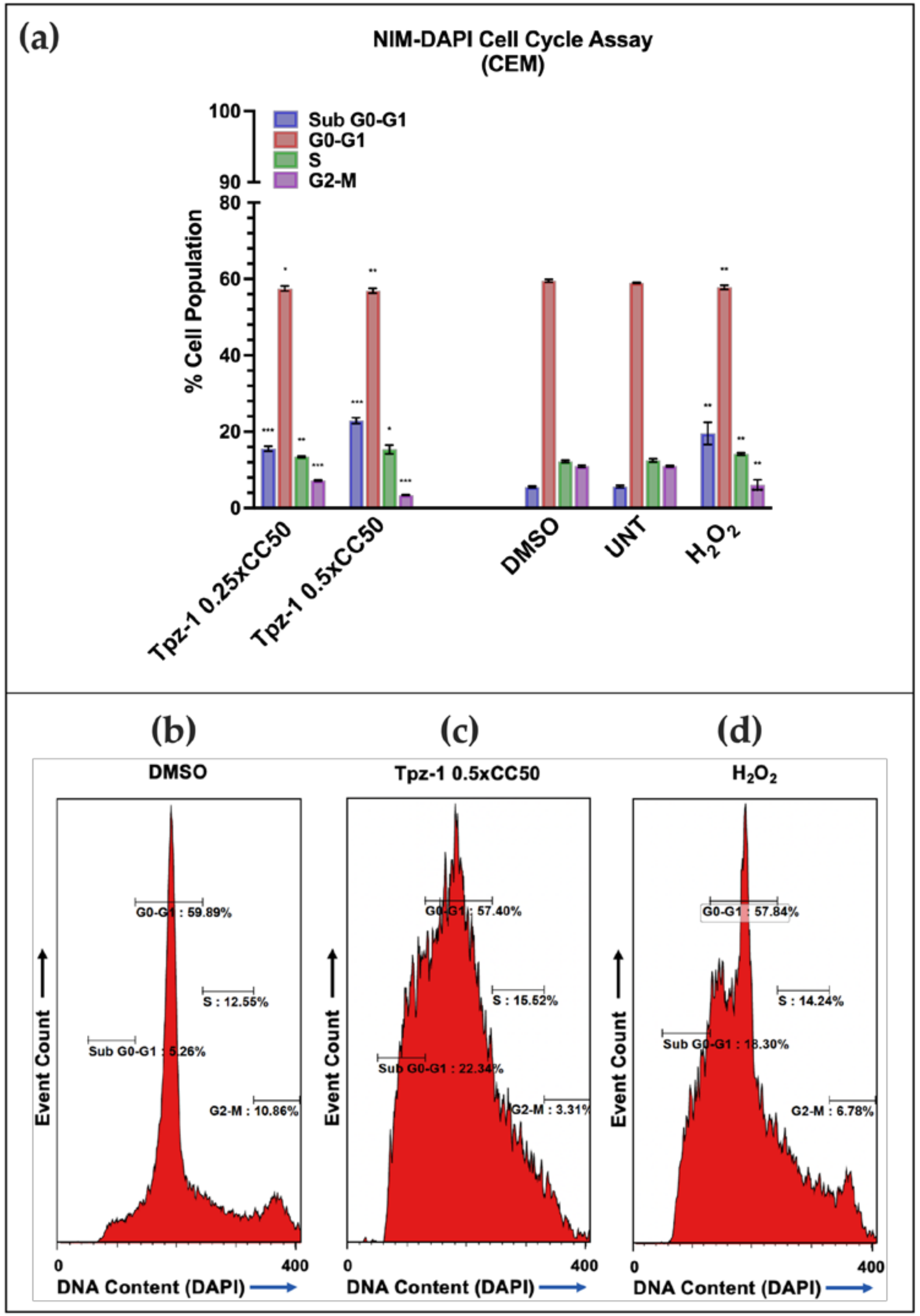
(a)
NIM-DAPI Cell Cycle Assay
(CEM)
Sub G0-G1
G0-G1
S
G2-M
% Cell Population
Tpz-1 0.25xCC50
Tpz-1 0.5xCC50
DMSO
UNT
H2O2
(b)
(c)
(d)
DMSO
Tpz-1 0.5xCC50
H2O2
G0-G1 : 59.89%
S : 12.55%
Sub G0-G1 : 5.26%
G2-M : 10.86%
G0-G1 : 57.40%
S : 15.52%
Sub G0-G1 : 22.34%
G2-M : 3.31%
G0-G1 : 57.84%
S : 14.24%
Sub G0-G1 : 18.30%
G2-M : 6.78%
Event Count
DNA Content (DAPI)
